# A comprehensive review on *Moringa oleifera* nanoparticles: importance of polyphenols in nanoparticle synthesis, nanoparticle efficacy and their applications

**DOI:** 10.1186/s12951-024-02332-8

**Published:** 2024-02-19

**Authors:** Haribalan Perumalsamy, Sri Renukadevi Balusamy, Johan Sukweenadhi, Sagnik Nag, Davoodbasha MubarakAli, Mohamed El-Agamy Farh, Hari Vijay, Shadi Rahimi

**Affiliations:** 1 Institute for Next Generation Material Design, Hanyang University, Seoul, Republic of Korea; 2 Center for Creative Convergence Education, Hanyang University, Seoul, Republic of Korea; 3 Department of Chemistry, College of Natural Sciences, Hanyang University, Seoul, Republic of Korea; 4 Department of Food Science and Biotechnology, Sejong University, Gwangjin-Gu, Seoul, 05006 Republic of Korea; 5 Faculty of Biotechnology, University of Surabaya, Surabaya, 60293 Indonesia; 6 Pharmacology Unit, Jeffrey Cheah School of Medicine and Health Sciences (JCSMHS), Monash University Malaysia, 47500 Bandar Sunway, Selangor Darul Ehsan Malaysia; 7 School of Life Sciences, B.S. Abdur Rahman Crescent Institute of Science and Technology, Chennai, Tamil Nadu India; 8 Department of Radiation Oncology, College of Medicine, Yonsei University, Seoul, South Korea; 9 Global Health Research, Saveetha Medical College, Saveetha Institute of Medical and Technical Sciences, Chennai, India; 10 Division of Systems and Synthetic Biology, Department of Life Sciences, Chalmers University of Technology, Gothenburg, Sweden

**Keywords:** Moringa nanoparticles, Metal nanoparticles, Green synthesis, Secondary metabolites, Application of Moringa nanoparticles, Critical parameters

## Abstract

*Moringa oleifera* is one of the popular functional foods that has been tremendously exploited for synthesis of a vast majority of metal nanoparticles (NPs). The diverse secondary metabolites present in this plant turn it into a green tool for synthesis of different NPs with various biological activities. In this review, we discussed different types of NPs including silver, gold, titanium oxide, iron oxide, and zinc oxide NPs produced from the extract of different parts of *M. oleifera.* Different parts of *M. oleifera* take a role as the reducing, stabilizing, capping agent, and depending on the source of extract, the color of solution changes within NP synthesis. We highlighted the role of polyphenols in the synthesis of NPs among major constituents of *M. oleifera* extract. The different synthesis methods that could lead to the formation of various sizes and shapes of NPs and play crucial role in biomedical application were critically discussed. We further debated the mechanism of interaction of NPs with various sizes and shapes with the cells, and further their clearance from the body. The application of NPs made from *M. oleifera* extract as anticancer, antimicrobial, wound healing, and water treatment agent were also discussed. Small NPs show better antimicrobial activity, while they can be easily cleared from the body through the kidney. In contrast, large NPs are taken by the mono nuclear phagocyte system (MPS) cells. In case of shape, the NPs with spherical shape penetrate into the bacteria, and show stronger antibacterial activity compared to the NPs with other shapes. Finally, this review aims to correlate the key characteristics of NPs made from *M. oleifera* extract, such as size and shape, to their interactions with the cells for designing and engineering them for bio-applications and especially for therapeutic purposes.

**Graphical Abstract:**

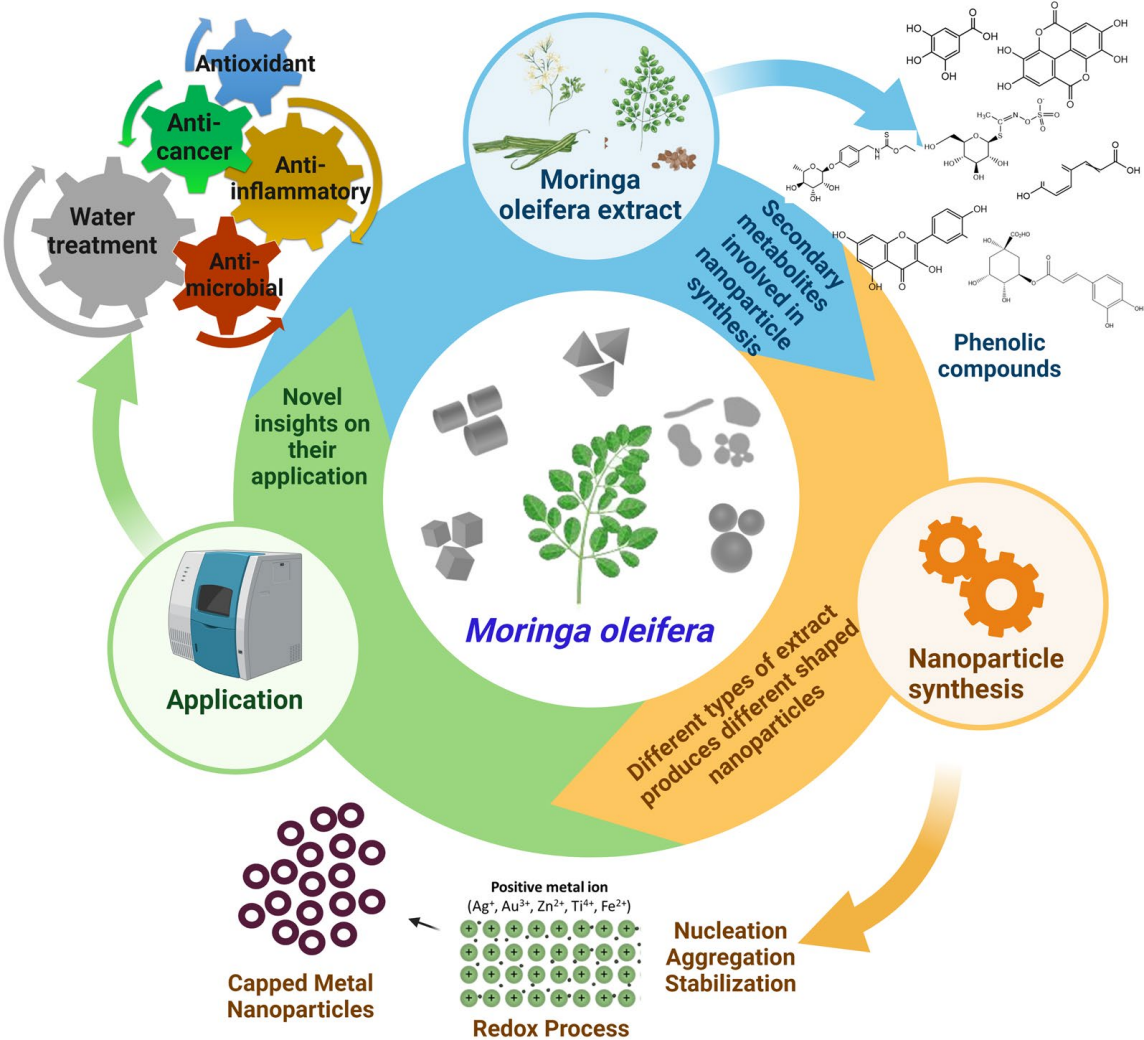

## Introduction

Plant-based products have recently attracted a great interest in multiple applications including food production, health products, and bioremediations due to their sustainability, low cost, and less adverse effects on human health [1, 2]. Plant-mediated nanoparticles (NPs) are one of these products which have an increasing demand over the chemically synthetized counterparts due to their minimalistic environmental footprint [3]. *M. oleifera* is one of the plants that has been tremendously exploited for synthesis of a vast majority of nanoparticles including silver, gold, bismuth, iron, ZnO, MgO, NiO, CuO, and the rare nanometal, CeO [4–18]. Details in respect to synthesis of these various nanometals using *M. oleifera* are summarized in Table 1.

**Table 1:** Synthesis, physiochemical properties, secondary metabolites, and applications of moringa nanoparticles

Metal NPs	Plant parts	Synthesis method	Secondary metabolites	Size (nm)	Shape	Zeta potential	Applications	References
Silver NPs (AgNPs)								
AgNPs	Leaves	Green	–	57	Spherical		Antimicrobial	[97]
AgNPs	Leaves (freeze dried)	Green	Flavones, terpenoids and polysaccharides	11	Spherical	–	Antibacterial	[15]
AgNPs	Leaves (fresh)	Green	Flavones, terpenoids and polysaccharides	9	Spherical	–	Antimicrobial	[15]
AgNPs	Leaves	Green	–	5–80	Spherical	–	Optical limiting	[57]
AgNPs	Leaves	Green	–	96.72	Spherical	–	–	[58]
AgNPs	Leaves	Green	Phenolic compounds	5–10	Spherical	–	Antioxidant, cytotoxicity	[33]
AgNPs	Leaves	Green	Alkaloids, phytosterol, saponins, terpenoids	–	–	–	Antibacterial	[58]
AgNPs	Leaves	Green	Terpenoids, flavonoids and polysaccharides	116.2 d.nm	Spherical or Polygonal	–	Antioxidant, Reduction in size of leishmaniasis	[34]
AgNPs	Leaves	Green	–	1–56.9, 2–448.1, 3–4705	–	–	Antimicrobial	[98]
AgNPs	Leaves	Green	–	32	Cubic	–	–	[59]
AgNPs	Leaves	Green	–	20–40	Spherical	–	Antimicrobial	[92]
AgNPs	Leaves	Green	–	10–100	Round	–	Cytotoxicity	[13]
AgNPs	Leaves	Green	–	30	Spherical	–	Antimicrobial	[63]
AgNPs	Leaves	Green	Flavonoid-kaempferol, phenolic-chlorogenic, tannins	11–14.3	Rod	–	Antibacterial	[35]
AgNPs	Leaves	Green	Flavonoids, phenolic compounds	50	Spherical and rectangular	–	Cytotoxicity	[36]
AgNPs	Leaves	Green	–	95.12	Spherical	–	–	[160]
AgNPs	Stem bark	Green	Terpenoids, flavonoids and polysaccharides	40	Spherical and Pentagon	–	Anticancer	[37]
AgNPs	Stem bark	Green	–	–	–	–	–	[60]
AgNPs	Seeds	Green	Chlorophyll a, b; carotenoids, phenol, flavonoids	15.5 ± 4.2	Spherical	–	Immobilization, modulators of heavy metals	[161]
AgNPs	Crude gum	Green	–	60	–	–	Antibacterial	[10]
AgNPs	Crude gum	Green	–	9	Spherical	–	Antimicrobial	[162]
AgNPs	Crude gum	Green	–	50	–	–	Antibacterial	[10]
AgNPs	Flower	Green	–	273.98	Spherical	–	–	[58]
AgNPs	Flower	Green	Flavonoids and phenolic compounds	8	Spherical	–	Antibacterial	[4]
AgNPs	Leaves	Green	–	–	–	−27	Anticancer	[92]
AgNPs/AuNPs	Leaves	Green	–	11–25	Hexagonal, triangular and spherical	−36.7	Anticancer	[92]
Gold nanoparticles (AuNPs)								
AuNPs	Leaves	Green	–	–	–	−36.9	Anticancer	[92]
AuNPs	Leaves	Green	–	10–20	Spherical	−25.3	Antiproliferative, apoptosis	[65]
AuNPs	Leaves	Green	–	20–60	Spherical	−24.09	Stability	[67]
AuNPs	Pod	Green	Alkaloid, terpenoids, flavonoid, phenols, tannins, saponins quinines, proteins	40–80	Distinct	–	Antibacterial, hepatoprotective	[64]
AuNPs	Gum	Green	–	5–45	Spherical	–	DNA protection	[66]
Zinc oxide nanoparticles (ZnONPs)								
ZnONPs	Leaves	Green		52	Spherical	–	Photocatalytic, antibacterial	[83]
ZnONPs	Leaves	Green	Vitamins, flavonoids, and phenolic acids	12.27–30.51	–	–	Electrochemical activity	[42]
ZnONPs	Leaves	Green	–	10.81	Wurtzite	–	–	[85]
ZnONPs	Leaves	Green	–	17.8	Cubic	–	–	[85]
ZnONPs	Leaves	Green	Phenols and Amines	10	Irregular	–	Effective adsorption	[86]
ZnONPs	Leaves	Green	Phenolic compounds	25	Sphere	–	Antibacterial	[38]
ZnONPs	Flower	Green	–	13.2	–	–	Effective chelating agent	[84]
ZnONPs	Seed	Green	–	13.2	–	–	Effective chelating agent	[84]
ZnONPs	Crude gum	Green	–	60	–	–	Antibacterial	[10]
ZnONPs	Crude gum	Green	–	60	–	–	Antibacterial	[10]
Iron oxide nanoparticles (FeONPs)								
FeONPs	Leaves	Green	Polyphenols	30.5	Non-spherical	–	Antioxidant, antibacterial	[78]
FeONPs	Leaves	Green	–	37.3	Non-spherical	–	Antioxidant, antibacterial, thermal properties	[78]
FeONPs	Leaves	Green	–	82 ± 7	Spherical rod	–	Antibacterial, photocatalytic activity	[75]
FeONPs	Leaves	Green	–	15.01 ± 6.03	Rod	–	Antibacterial	[76]
Titanium oxide nanoparticles (TiO_2_NPs)								
TiO_2_NPs	Leaves	Green	–	12	Tetragonal	–	–	[69]
TiO_2_NPs	Leaves	Green	–	100	Spherical	–	Wound healing	[70]
TiO_2_NPs	Seeds	Green	–	90	Spherical	–	Apoptosis and anti-inflammatory	[54]

**Fig. 1:**
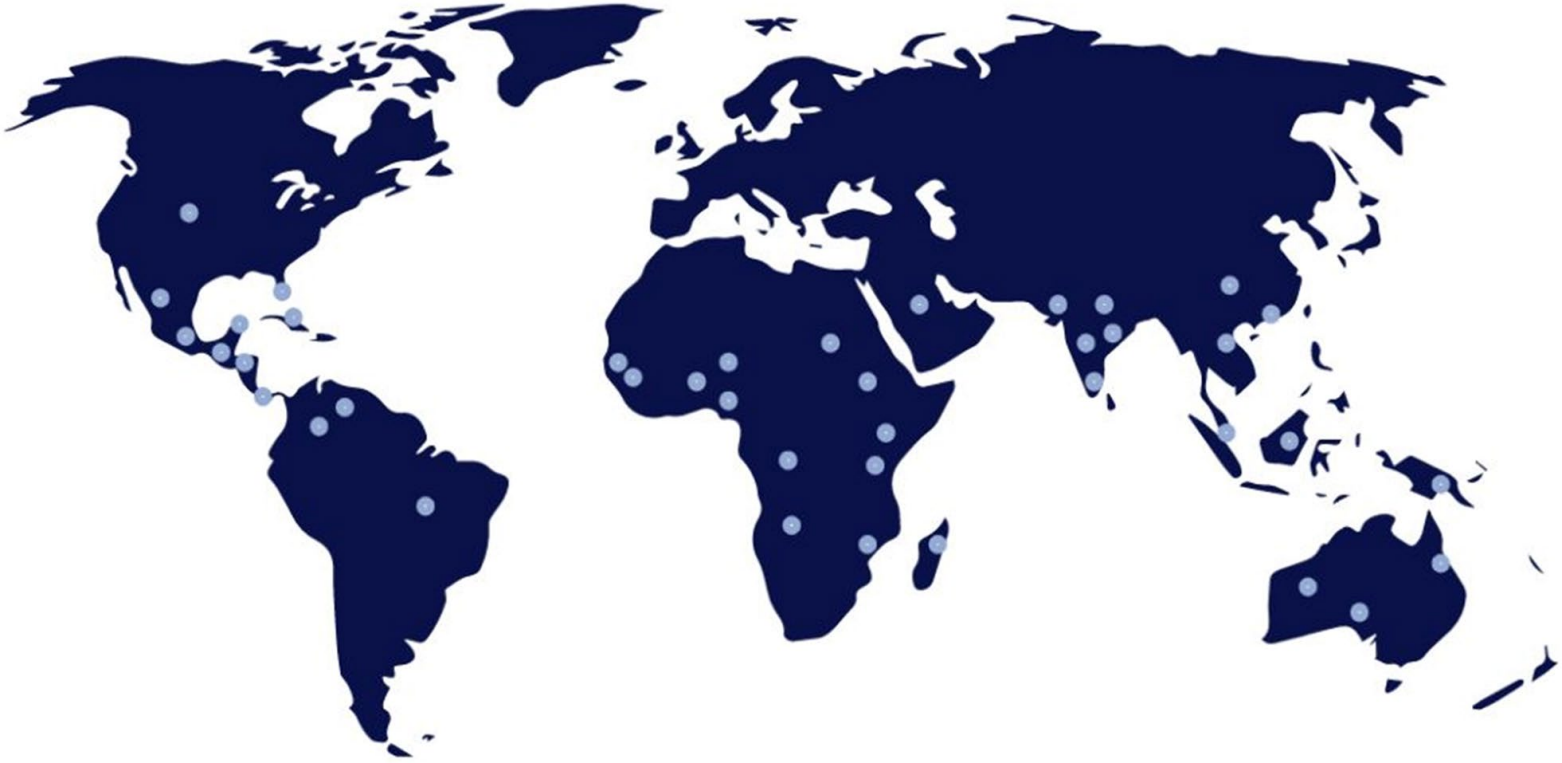
Geographical distribution of *M. oleifera*. *M. oleifera* is native to Asia and Africa, but grown in many other nations, as shown. Source: https://www.cabi.org/isc/datasheet/34868

*M. oleifera* is a popular species of Genus Moringa that has been characterized by fast growth and harsh condition adaptation [19, 20]. Figure 1 depicts the global distribution of *M. oleifera*. The tree natively exists across a wide range of Asian and African countries including India, Sri Lanka, Pakistan, Bangladesh, Afghanistan, Madagascar, and Arabian Peninsula [21]. The *M. oleifera* had been used by ancient Egyptians in cosmetics and for skin treatment. Also, it had been acknowledged by Romans and Greeks [22]. In time being, various parts of the plant were broadly used in several environmental, industrial, dietary supplements, and medical applications [23–25]. For instance, seed powder is used in the so-called coagulation-flocculation processes for cleaning drinking water from environmental contaminants such as pesticides, dyes, and pharmaceuticals [26, 27]. Also, different parts of the plant, including seeds, flowers, and leaves are good nutritious source of protein, lipids, vitamins, beta-carotene, minerals, and other nutrients, which are being exploited in food technology [24, 28–31]. The leaves also, besides the nutritious value, contain a large variety of secondary metabolites that exert anti-microbial, antioxidant, growth, and immune enhancing activities making the plant a potential alternative dietary supplement to livestock and poultry for organic meat and egg production, which has received a great public demand in recent years [32]. These constituents also exert pharmaceutical actions such as DNA protection, anti-cancer, anti-diabetic, anti-inflammatory, anti-ulcer, cardiovascular, anti-allergic, analgesic, wound healing, and fever relieving activities [23]. With all these great benefits, it comes as no surprise why *M. oleifera* is called “The Miracle tree” or “nature gift” [22, 28, 29].

**Fig. 2:**
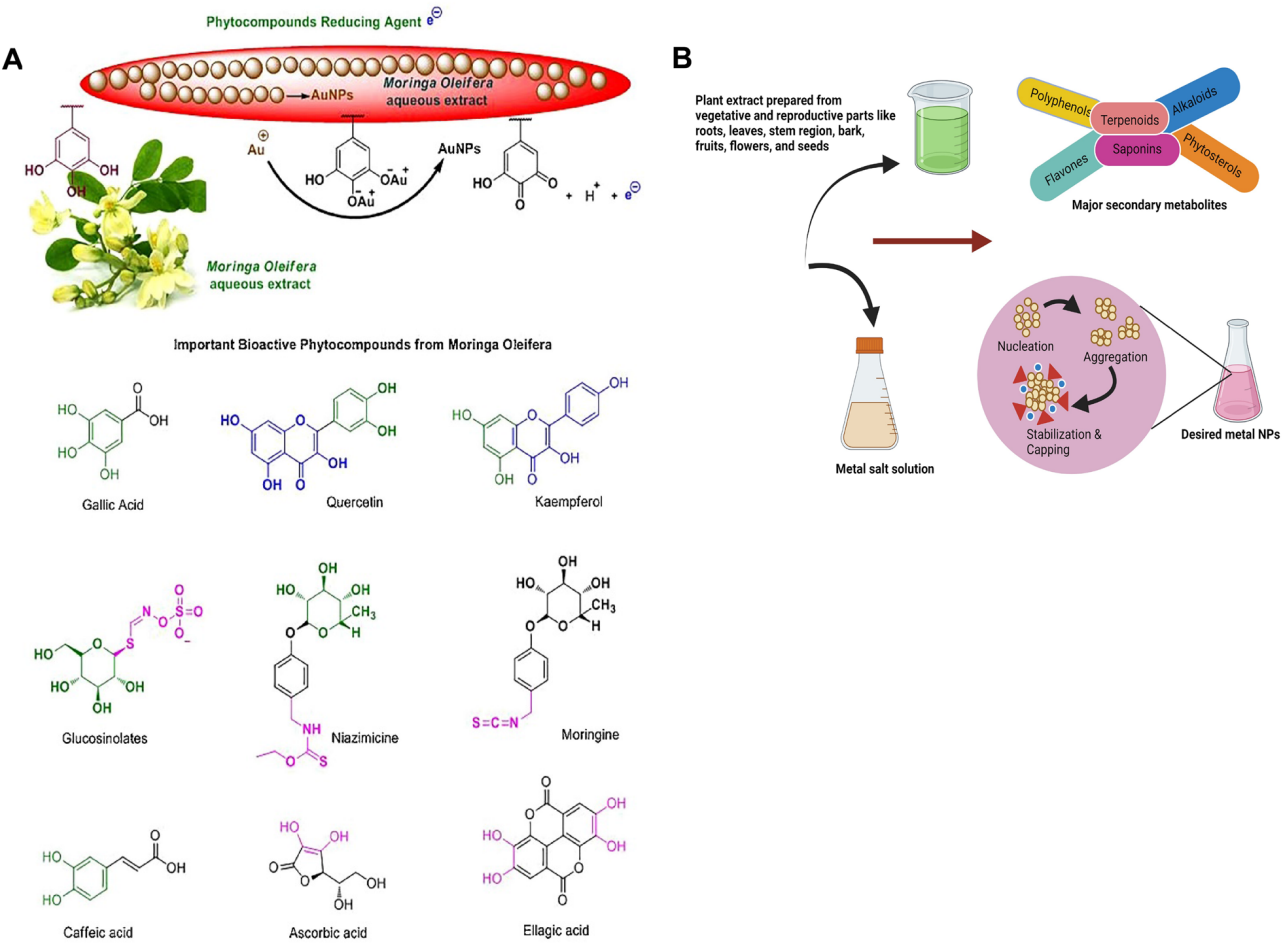
Metal NPs synthesis using various parts of *M. oleifera* extract. **A** Mechanism of gold NPs formation and bioactive compounds from *M. oleifera*. The original image was adapted with permission from Ref. [157]. 2023, Shadi Rahimi. **B** Significant secondary metabolites of *M. oleifera* involved in synthesis of metal NPs were highlighted

*M. oleifera* bioactive phytochemical profile includes quinine, saponins, flavonoids, tannin, steroids, glycosides, niazinins, niaziminins and many others [23] (Fig. 2A). This large diversity of secondary metabolites has drawn the attention towards *M. oleifera* as a green tool for synthesis of different NPs that exhibit biological activities. In some literature, it has been experimentally proven that secondary metabolites of *M. oleifera* can mediate synthesis of NPs with remarkable activities. For example, flavonoids, phenolics, polysaccharides, and terpenoids of the plant leaves, flowers, stem, peel, and pods are found to be the key players in synthesis of diverse NPs with variable sizes and shapes. The synthesized NPs showed a multiplicity of marveling biological activities including antibacterial, antioxidant, and cytotoxic and anticancer [4, 15, 33–41]. Owing to this, it is believed that the hidden power behind the biomedically active NPs is due to the plant part extracts utilized for the synthesis [4, 5, 10, 13, 42]. Besides biomedical applications, NPs synthesized by *M. oleifera* have also applications such as water decontamination via exerting antimicrobial and chelating activities [43].

Due to the growth flexibility, cultivation, and medicinal properties of *M. oleifera,* it has been adopted in many countries across the globe for industrial purposes [22]. Now, the global market size of *M. oleifera* products was estimated to be 5000 million USD in 2019. With increasing global demand, global market value is expected to reach 8400 million USD by 2026 [44]. Such exponential market increase emphasizes the necessity of learning all tales related to *M. oleifera* and its applications. A pool of literature reviews has discussed the utilization of *M. oleifera* extracts in medicine, industry, and environment sustainability, but few literatures have thoroughly presented the extract-based NPs synthesis, secondary metabolites (SMs) part in the synthesis process, and the biotechnological impact of synthesized NPs. Owing to overgrowing population across the world, reduced farming land, poor bioavailability of *M. oleifera* finds the way for the instigation of *M. oleifera* mediated synthesis positively for better future. Hence, our review will focus on exploitation of *M. oleifera* extracts in NPs synthesis, how *M. oleifera* secondary metabolites could be the driving force for NP synthesis, critical factors involved for NP synthesis, their applications, and the future prospects in this area.

## Green synthesis of metallic nanoparticles using various extracts of *M. oleifera*

In its entirety, the process of biosynthesizing metal nanoparticles with Moringa plant extracts comprises three primary stages: (1) the initiation phase, wherein the reduction of metal ions and the nucleation of reduced metal atoms transpire; (2) the growth phase, during which the aggregation of initially formed nanoparticles results in the spontaneous formation of larger particles (achieved through heterogeneous nucleation and growth, along with subsequent reduction of metal ions), concomitant with an elevation in the thermodynamic stability of the nanoparticles; and (3) the termination phase, dictating the ultimate morphology of the nanoparticles [45–50]. During the termination phase, nanoparticles assume the most energetically favorable configuration, and this step is significantly affected by the moringa plant extract's capacity to stabilize metal nanoparticles. For instance, nanotriangles, characterized by high surface energy, exhibit reduced stability. In the absence of adequate stability support within specific extracts, nanotriangles tend to adopt a more stable morphology, such as a truncated triangle, in an effort to minimize Gibbs free energy [51].

When a metallic salt undergoes dissociation into cations and anions, the cations undergo saturation to create hydroxyl complexes. Following the supersaturation of hydroxyl complexes, the growth of metal crystallites with oxygen species commences, leading to the development of crystalline planes with varying energy levels. The provision of heat assumes a pivotal role in supplying energy to the reaction system. This process persists until the activation of the capping agent derived from moringa plant extracts, ultimately halt the growth of high-energy atomic planes. Consequently, specific types of NPs are formed [52]. Broadly speaking, during the synthesis, the green reducing agents contribute electrons to the metal ions, thereby converting them into nanoparticles. The amine groups present in proteins and other significant secondary metabolites, along with the hydroxyl and carboxyl groups found in moringa polyphenols and amino acids, as well as the hydroxyl groups within polysaccharides and carboxyl groups in organic acids, play a crucial role in chelating metal ions. Through this mechanism, these functional groups on the secondary metabolites from the moringa phyto-extract catalyze the formation of metallic NPs [51, 53].

Silver (Ag), gold (Au), titanium oxide (TiO_2_), iron oxide (FeO), and zinc oxide (ZnO) NPs produced from the extract of different parts of *M. oleifera* are shown in Fig. 2B and are described as following.

### Silver nanoparticles (AgNPs)

Compared to physical and chemical synthesis, biological synthesis using *M. oleifera* is said to be clean, easy, non-toxic, and environmentally friendly, which is pH, temperature and time dependent NP synthesis [54]. Different parts of *M. oleifera* plant including flower, leaf, seed, and stem were used for silver nanoparticle (AgNPs) synthesis as follows. The spherically monodispersed AgNPs prepared using Moringa flower extract showed average crystalline size of 8 nm with increased inhibition of bacterial growth such as *Klebsiella pneumoniae* and *Staphyllococcus aureus* [4]. Because of the reduction of silver ion and the creation of AgNPs, the biofabrication of AgNPs using *M. oleifera* leaf extract produced a reddish brown color [55]. AgNPs produced from *M. oleifera* leaf extracts displayed antibacterial activities when exposed to sunlight as the principal source of energy [15].

Biosynthesis of colloidal shaped AgNPs were synthesized by reduction method using *M. oleifera* leaf extract as a reducing agent, and exhibited anti-microbial properties [15]. *M. oleifera* AgNPs were synthesized using a clean, non-toxic and environmentally friendly approach, which possessed significant biological activity against diarrhea bacterial strains such as *E. coli*, and *S. aureus* [56]. AgNPs using fresh leaves of *M. oleifera* as a reducing and stabilizing agent, produced polydisperse nanostructures such as irregular contours, nanorods, triangles etc., when leaves were cut finely and boiled for 5 min in water and filtered [57]. Green synthesis was used to produce AgNPs from *M. oleifera* leaf extract, and phytochemicals such as reducing sugar, flavonoids, and phenolic compounds found in the extract are responsible for the conversion of silver ion to metallic silver, resulting in spherical and rectangular NPs [36]. A multivariate optimization employed in the synthesis of AgNPs using *M. oleifera* stem bark, leaves and flower water extracts showed spherical shaped nanostructures of 95.12 nm, 96.72 nm, and 273.98 nm, respectively [58]. A facile and dependable green production of AgNPs from moringa leaf aqueous extract yielded spherical nanostructures [13]. A simple, quick, and environmentally friendly synthesis of stable AgNPs utilizing *M. oleifera* leaf extract was developed. The resulting crystalline AgNPs feature centered-cubic shape and an average size of 32 nm [59]. *M. oleifera* leaves and seeds were employed for the simple production of AgNPs by reducing AgNO_3_ to tannic acid under vigorous shaking with the stabilizer sodium citrate until a dark gray colloidal solution was produced [33]. The seeds of *M. oleifera* were used for the synthesis of AgNPs, where colorless solution was turned into brown color indicating the formation of NPs [60]. *M. oleifera* stem bark extract was used for stable formation of AgNPs which can be used for the treatment of cancer [37]. AgNPs produced biologically by the reducing action of secondary metabolites found in the stem of *M. oleifera*, exhibited great potential for rapid synthesis of NP with antibacterial activity [61].

There are several simple and eco-friendly methods used for AgNPs synthesis using *M. oleifera.* In a work, *M. oleifera* leaf extract was employed to create AgNPs in a simple and environmentally friendly manner. This method is considered green chemistry because it uses natural plant extracts instead of harmful chemicals, making it more environmentally friendly. The process was found to be proficient, meaning that it was effective in producing high-quality AgNPs, which had a uniform size distribution, good stability, and high purity [62]. A non-toxic, eco-friendly, simple, and efficient synthesis of AgNPs were synthesized in cold condition using aqueous extract of *M. oleifera* leaves extract [63]. A biogenic synthesis of polyethylene glycol (PEG) induced AgNPs formation using organic compounds of *M. oleifera* aqueous extract is considered as benign, facile, biocompatible, cost-effective, and eco-friendly method [35]. Using *M. oleifera*, which contains flavonoid, tannin, and phenol phytochemicals as the capping and reducing agent to reduce the silver ions to AgNPs, an easy one-step green technique for AgNP synthesis was developed [35]. *M. oleifera* gum-based AgNPs were successfully synthesized through ecofriendly, sustainable, economical and easy to use green synthesis process [10].

**Fig. 3:**
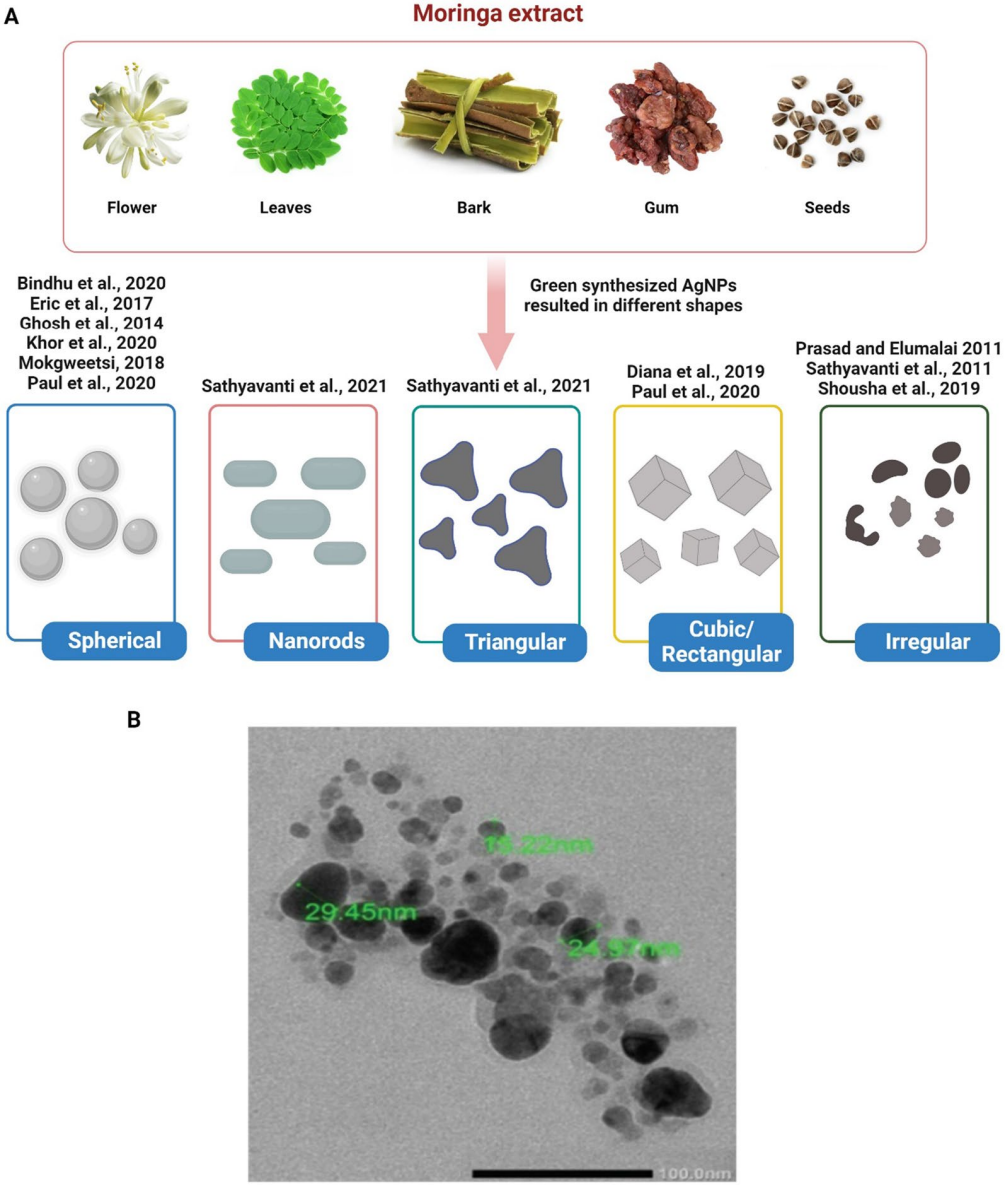
**A** Different *M. oleifera* extract produces AgNPs in various shapes. **B** TEM image of AgNPs biosynthesized with *M. oleifera* leaf extract. The original image was adapted from Ref. [158]

In conclusion, different *M. oleifera* parts, such as the flower, leaf, seed, and stem, were employed for the synthesis of AgNPs, and depending on the synthesis technique, AgNPs of various shapes and sizes could be produced (Fig. 3).

### Gold nanoparticles (AuNPs)

The synthesis of gold nanoparticles (AuNPs) using pods of *M. oleifera* uses secondary metabolites for NP synthesis. Compared to the ethanol and chloroform extracts, aqueous extracts of pods were highly suitable for AuNP synthesis because of the presence of saponins, flavonoids, tannins, steroids, and alkaloids [64]. One-pot green synthesis of AuNPs using leaves of *M. oleifera* resulted in red-brown color formation within few seconds of mixing leaf extract with the HAuCl_4_ solution. This is due to the excitation of surface plasmon vibrations in AuNPs [65]. A simple, easy to handle, biocompatible AuNPs synthesized using gum extracts of *M. oleifera* resulted in the color change from pale yellow to ruby red color formation due to the action of gum extract as the reducing and stabilizing agent for AuNPs synthesis. The presence of leaf proteins and metabolites in the gum extract of *M. oleifera* is what causes the decrease of gold ions and stabilization of AuNPs [66]. An eco-friendly approach was to synthesize AuNPs by chloroauric acid from *M. oleifera* leaf extract as the reducing agent, thus resulting in the stable and spherical NP production [67]. The creation of pink hue in the stable AuNPs from aqueous 1 M chloroauric acid and *M. oleifera* flower aqueous extract was attributed to the plasmon vibrations in the AuNPs being excited by a redox reaction mechanism. The capping agents and molecules such as aliphatic and trace aromatic compounds present in the flowers of *M. oleifera* are responsible for the AuNPs formation [68].

**Fig. 4:**
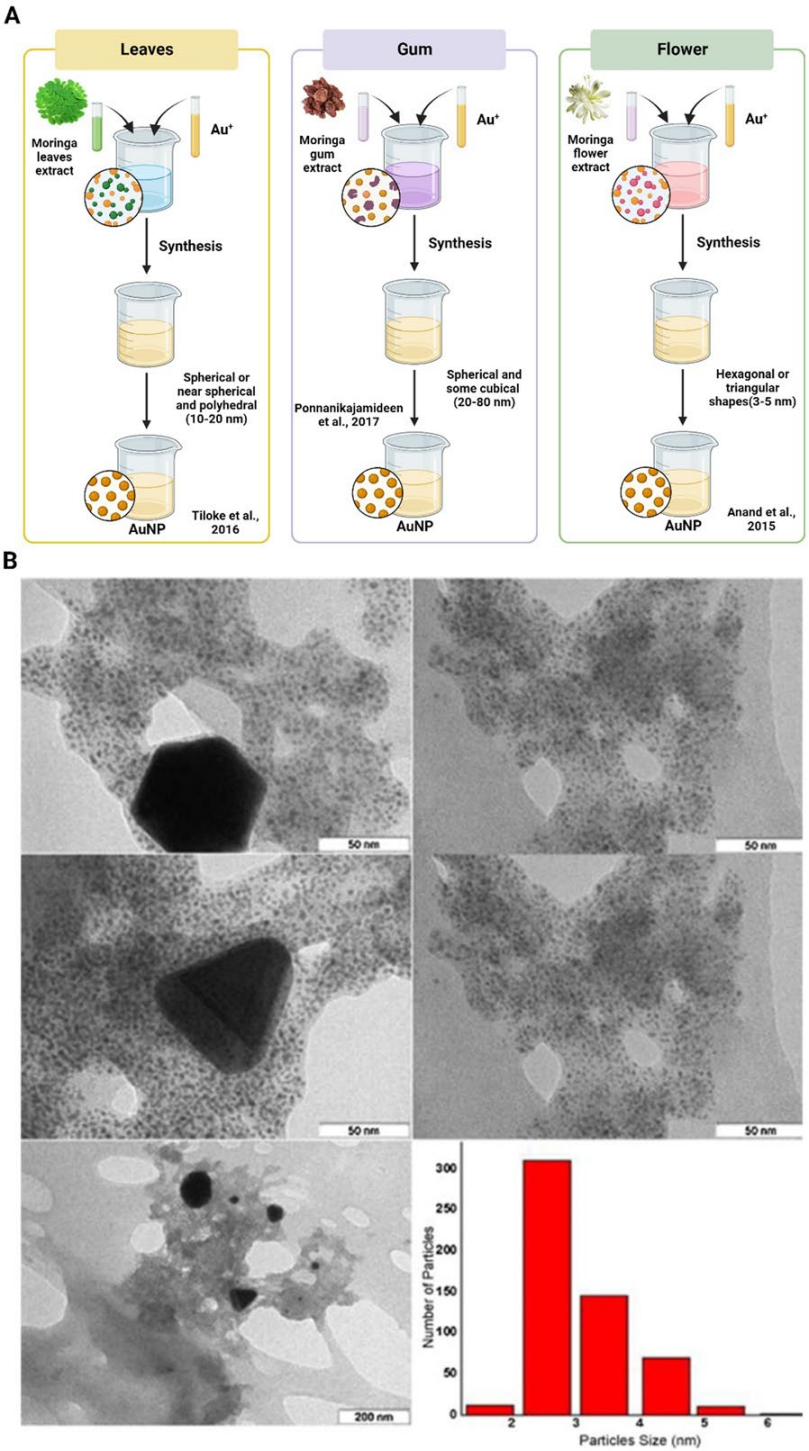
**A** AuNPs synthesis using different *M. oleifera* extracts as reducing agents. Different *M. oleifera* extracts as the reducing agents caused various color change in AuNPs. **B** TEM images and size distribution of AuNPs biosynthesized by aqueous flower extracts of *M. oleifera*. The original image was adapted with permission from Ref. [68]. 2023, Shadi Rahimi

In summary, AuNPs could be synthesized using pods, leaves, flower, and gum extracts of *M. oleifera* and based on the extract source, the AuNP solutions with different colors are produced (Fig. 4).

### Titanium oxide nanoparticles (TiO_2_ NPs)

Useful in a wide range of applications, the biosynthesis of titanium oxide nanoparticles (TiO_2_NPs) using Moringa extract is simple, affordable, time-saving, and environmentally benign [69]. The environmentally friendly green chemistry that produces NPs using leaf extract will improve economic viability and sustainable management. Effective TiO_2_NPs synthesis was achieved either with aqueous extract [70–73] or ethanolic extract [69] of *M. oleifera* under various condition. Most of previous results reported using leaves of Moringa [69–72], but there are also those who used seeds [54] and sticks [73], to make TiO_2_ NPs.

Previous research used ethanolic leaf extract of Moringa to react with titanium tetra isopropoxide (TTIP) under continuous stirring at 50 °C for 4 h, resulting in tetragonal TiO_2_ NPs with an average size of 12 nm [69]. Sivaranjani [70]and Philominathan et al., [70] mixed an aqueous leaf extract of Moringa with a titanium dioxide solution, leading to the formation of spherical TiO_2_NPs with an average size of 100 nm. Kandeil et al. [54] reported similar characteristics of TiO_2_NPs produced by the high-energy ball mills method using Moringa seed extract. Pushpamalini et al. [71] employed a mixture of Moringa aqueous leaf extract, TTIP, and double distilled water to synthesize spherical TiO_2_NPs with an average size of 6.6 nm [71].

Umekar et al. [73] prepared phytoreduced graphene oxide-titanium dioxide nanocomposites (r-GO/TiO_2_ NCs) using synthesized graphene oxide, TiO_2_NPs, and *M. oleifera* stick extract through a precipitation technique. Continuous agitation for 8 h at room temperature was required to achieve a uniform dispersion and anchoring of TiO_2_NPs onto the graphene oxide sheet. Furthermore, Satti et al. [72] reported the collection of irregular-shaped TiO_2_NPs with sizes ranging from 20 to 100 nm after continuous stirring of Moringa plant aqueous extract with a TiO_2_ salt solution for 24 h at room temperature.

**Fig. 5:**
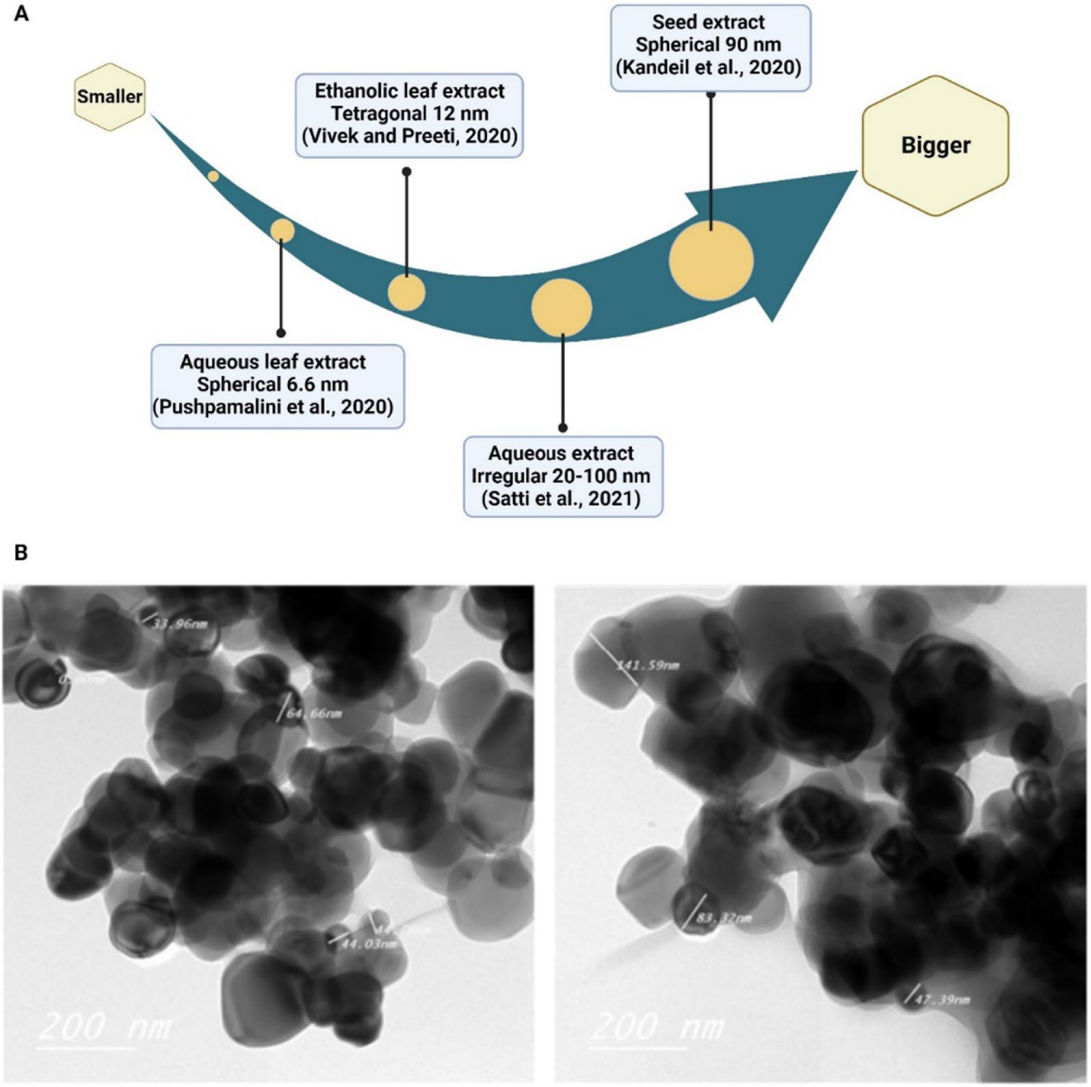
**A** Different *M. oleifera* extract produced TiO_2_ NPs in various sizes. **B** Spherical TiO2-NPs with a homogeneous nanometric size distribution of 90 nm (range 40–140 nm). The original image was adapted with permission from Ref. [54]. 2023, Shadi Rahimi

In general, leaves, seeds, and sticks of *M. oleifera* were used for TiO_2_ NP synthesis. TTIP and titanium dioxide solution were used for TiO_2_NP synthesis at various shapes and sizes (Fig. 5).

### Iron oxide nanoparticles (FeONPs)

The primary forms of iron oxide (FeO), one of the functional magnetic NPs, are magnetite (Fe_3_O_4_), hematite (α-Fe_2_O_3_), and maghemite (γ-Fe_2_O_3_). Iron oxide nanoparticles (FeO-NPs), which have a variety of uses including antimicrobial food coatings, catalysis and cosmetics, have received a great deal of attention due to their special qualities and applications [74]. Several reports stated the successful production of iron or iron oxide NPs using Moringa leaves [43, 75–77], fruit [78], seeds extract [27, 43, 79] and its oils [80].

FeONPs were synthesized using a cost-effective method utilizing *M. oleifera* for biomedical applications. This means that it does not require expensive equipment or harmful chemicals, which can significantly reduce the cost of production. Additionally, *M. oleifera* is an easily accessible and abundant plant in many low and middle-income countries, making it a cost-effective source for the synthesis of these NP [81]. FeONPs synthesized using *M. oleifera* have potential biomedical applications, particularly in targeted drug delivery systems. For more effective and efficient medication delivery, these NPs can be functionalized with certain ligands or antibodies to target cells or tissues in the body. Additionally, FeONPs possess magnetic properties that can be applied to MRI and hyperthermia therapy for the treatment of cancer [81]. Aqueous extracts of the fruit and leaves of *M. oleifera* were used to create iron nanoparticles (NPs) in a green manner (MOL). MOF-Fe and MOL-Fe have erratic shapes, as seen in TEM and SEM photos (particle size of 45 nm). By adding 0.5 M of Fe-salt dropwise to the aqueous plant extract in a sonicated reactor, biogenic Fe NPs were created. Iron was present, as shown by the formation of NPs with a black hue. Fe NPs displayed peaks about 400 nm, while the Fe-salt solution displayed a peak around 300 nm. The identical spectra at 300 nm were also seen in the nanosuspension of Fe NPs, confirming the inclusion of Fe NPs in the suspension [78]. Meanwhile, Katata-Seru et al. (2018) reported that Fe NPs based on MOL and aqueous extracts of *M. oleifera* seeds (MOS) exhibited absorbance at 210 and 240 nm in the UV–Vis spectrum. The size range was determined to be between 2.6 and 6.2 nm, and HRTEM (High-Resolution Transmission Electron Microscopy) revealed that MOS-FeNPs are spherically structured with thick surface layers. This could be because the layer of MOS has thickened. The MOL-FeNPs' spherical form and diameter of 3.4 and 7.4 nm were confirmed by HRTEM analysis [43]. Another report found that adding 20 mL of PMC (*Psidium guajava*—*M. oleifera* composite) extract to the solution of FeCl_3_ at a volume ratio of 8:2 (V/V) for 2 min resulted in a color change from orange to dark brown, confirming the synthesis of PMC-NPs. After another hour of stirring, the homogenous solution was transferred to a hot air oven set at 100 °C for 24 h. The generated NPs have a non-uniform rod-like morphology [75]. In the other hand, the mixture's color changes from green to black, making it easy to see that FeO NPs are forming. FeO NPs produced from the leaf extract *of M. oleifera* showed spherical agglomerations with an average diameter of under 100 nm [77].

Without using organic solvents, iron oxide (maghemite, γ-Fe_2_O_3_) NPs were made using a modified sol–gel approach and functionalized with Mo saline solution. By using the Scherrer equation, the average NP size was determined to be 15 nm. The average size determined by Scherrer's equation is corroborated by the average size seen in TEM micrographs (between 10 and 20 nm). The NPs have cubic shape [27]. Iron oxide nanorods (FeO-NRs) were reported to be produced biochemically from FeCl_3_ and *M. oleifera*. The formation of FeO-NRs is caused by the visible color changing. X-ray diffraction spectroscopy was used to observe the crystallinity of FeO-NRs, and the results revealed a pattern consistent with the spinel cubic lattice in the tetrahedral hematite structure. The size of the crystallite was estimated to be between 40 and 90 nm. FeO-NRs were found to have a rod-like shape, with an average particle size of 15.01 ± 6.03 nm [76].

The use of methyl ester of *M. oleifera* oil over Fe-Co impregnated Alumina support at 650 °C under N_2_ environment resulted in an eco-friendly green precursor for the production of multi-walled carbon nanotubes (MW CNTs). The diameters of as-synthesized nanotubes ranged from 19 to 22 nm. The interior diameters of the nanotubes ranged from 14 to 18 nm, whereas the exterior diameters of the produced nanotubes ranged from 42 to 50 nm [80]. In order to remediate surface water with high turbidity, the interaction of functionalized magnetic NPs with proteins from *M. oleifera* seeds was studied. At room temperature (25 ± 2 °C) in an environment of oxygen, Fe_3_O_4_ NPs were made utilizing the co-precipitation method in aqueous solution with ferric salts as the precursors. The functionalized nanoparticles were called MOFe_3_O_4_. For Fe_3_O_4_ and MOFe_3_O_4_, the Scherrer equation calculated the average crystallite sizes to be 17 nm and 35 nm, respectively [79]. The soluble proteins and other substances in the MO saline extract may be responsible for this increase in crystallite size following functionalization. According to Cheng et al. [82], ferromagnetic magnetite often has NPs with diameters smaller than 50 nm and a black coloring [82], and this has been observed in this study [79].

In summary, leaves, fruit, seed, and oil of *M. oleifera* were used for FeONPs. UV–Vis spectrum and the color change of solution after synthesis could affirm the formation of Fe NPs.

### Zinc oxide nanoparticles (ZnO)

The precipitation method was reported for synthesizing zinc oxide nanoparticles (ZnO NPs) using *M. oleifera* leaves extract as the natural precursor. The NPs have hexagonal wurtzite structure of an average grain size of 52 nm [83]. ZnO NPs with particle size ranging from 12.27 and 30.51 nm were effectively synthesized by the extract of *M. oleifera* leaves. It was discovered that annealing in air at 500 °C is essential for the manufacture of pure wurtzite ZnO phase [42]. Similar to this, ZnONPs were effectively produced employing a green method and an efficient chelating reduction/oxidizing agent—leaves from *M. oleifera*. According to Debby Scherrer's equation for ZnONPs, the average crystallite size was 10.81 nm [84]. In a different investigation, *M. oleifera* leaf extract was used to successfully produce ZnONPs. Cu was successfully doped into the ZnO lattice, as evidenced by all of the techniques used in the characterization [85]. Cu dopes the NPs and increases their effectiveness as an adsorbent for the removal of Congo red dye from aqueous solutions in the manufacture of ZnONPs using *M. oleifera* leaf extract. Congo red is adsorbed on both types of NPs with a pseudo-second-order kinetics, with the Cu-ZnONPs being shown to be more effective as an adsorbent than the undoped ZnONPs [86]. Another group studied the structural and optical differences between ZnONPs made from leaves and those made from different extracts. While the Tauc approximation yields direct band gap measurements are 3.25, 3.18, and 3.12 eV for ZnONPs synthesized by the leaves, seeds, and flowers, respectively, the calculated average crystallite sizes of ZnONPs are found to be 10.8, 13.9, and 13.2 nm. Regarding average crystallite size, smaller sizes are generally desirable as they can enhance the NPs properties such as surface area, reactivity, and optical behavior. When considering the direct band gap values, a larger band gap signifies a wider energy range where the material does not allow electron conduction. This can be advantageous in applications where the material needs to exhibit higher electrical resistivity or ultraviolet (UV) light absorption. Based on those factors, the ZnONPs from *M. oleifera* leaves has the best results [84]. A simple and eco-friendly synthesis of ZnONPs using root extract of *M. oleifera* resulted in the average NPs size of around ~ 25 nm with 2.5 of the peak width. The morphology of the particles was analyzed by TEM and showed that they were spherical in shape [38]. The synthesized NPs utilized crude of Moringa gum, were found to be about 60 nm and 50 nm for ZnONPs and AgNPs, respectively, which is considered an ideal size range for nanomaterials. The synthesized NPs exhibited absorption band in the range of 220–300 nm, a good Plasmon resonance band (PRB) of Ag and Zn NPs. On the other hand, the purified gum powder was found to have a much larger size. This difference in size can be attributed to the fact that during the synthesis process, the gum acts as a reducing and capping agent, which helps in controlling the size and morphology of the NPs. In contrast, the purified gum powder does not have such properties, which results in larger particle size [10].

In short, leaves, flowers, seeds, root, and crude gum of Moringa were used for ZnONP synthesis. Interestingly, the leave extract could produce smaller size ZnONP compared to flowers, and seeds extracts.

## Phytochemicals in metal NPs formation

The utilization of plant extracts provides advantages over other biosynthetic strategies, including accessibility, secure handling, and broad metabolite sustainability. Terpenoids, flavones, ketones, aldehydes, and amides are often the primary phytochemicals involved in the synthesis of TiO_2_NPs [69]. This is in synergy with reports of a smooth, green and one-pot synthesis method using Moringa leaf extract to synthesize TiO_2_NPs [70–72]. The ethanol extract of *M. oleifera* seeds showed 11 major peaks in the chromatogram of extract. The extract's primary chemical components include phenolic compounds (aceteugenol and eugenol), anti-inflammatory terpenes and sesquiterpenes (cadinese, humulene, copaene, and ocimene), fatty acids (trans-13-octadecenoic and hexadecanoic acid), and others that are not known to be present in any known quantities. As the reducing agent in the production of biomolecules such as proteins, carbohydrates, lipids, and nucleic acids, FeO-NPs and FeO-NRs' Fourier-transform infrared spectroscopy (FT-IR) spectra study revealed various vibrational bands of these molecules [54].

Many studies have used the FT-IR spectra of Moringa extracts and nanosuspensions to provide evidence for the green synthesis of Fe or FeONPs. The spectra show peaks that correspond to O–H bonds, which are present in polysaccharides, proteins, and polyphenols, as well as water molecules. Nanosuspensions refer to a type of nanoparticle formulation where the drug particles are suspended in a liquid medium, usually water, with the help of a stabilizer. The particle size of the drug in nanosuspensions is usually in the range of 10–1000 nm [87], C = O (carbonyl group), C–O and C–H stretching [43, 75, 76, 78]. Other ending vibration of C-N (of aromatic amines) and C–O–C functional groups were also observed [75]. The observed vibration bands below 600 cm^−1^ give birth to the stretching mode of the Fe–O bond [88], evidence of the successful immobilization of Moringa metabolites on the surface of the formed NPs [75]. XRD analysis could confirm the presence of hematite (α-Fe_2_O_3_) and ferric oxyhydroxide (FeOOH) in the Fe NPs [78]. Following the synthesis, there was a reduction in the extract's total phenolic concentration, which showed that the polyphenols were involved in the procedure [78].

The presence of distinct functional groups from diverse biomolecules in the leaves extract of *M. oleifera* plant that are responsible for the production and stability of ZnS nanostructures, was detected using FT-IR spectroscopy on the biosynthesized ZnSNPs. The presence of certain biomolecules in the 90 percent ethanolic extracts of *M.oleifera* leaves was discovered and quantified using the results of a study that used thin-layer chromatography densitometry. The average levels of the biomolecules crypto-chlorogenic acid, astragalin and isoquercetin identified in the study were 0.042%, 0.0634% and 0.0467% dry weight, respectively. When compared to the chemical synthesis approach, the biomolecules found in the leaves extract of *M. oleifera* plant acted as reducing and stabilizing agents, resulting in a 4 h reaction time. The measured negative zeta potential values of 45–55 mV confirm the biosynthesized NPs' remarkable stability over a 3-month timeframe. This demonstrates the innovative nature of the biosynthetic process for ZnSNP production [89].

For each of the primary family bioactive chemicals, a mechanism of ZnONP synthesis via the chemical interaction of Zinc nitrate precursor with *M. oleifera* bioactive compounds is proposed: vitamin supplements, flavonoids, and phenolic acids [42]. FT-IR analysis showed significant changes in the functional groups of various polyphenols present in the root extract [38]. The purified gum from the *M. oleifera* plant contained a variety of flavonoids, proteins, and carbohydrates, including D-galactose, D-xylose, D-glucuronic acid, D-mannose, L-arabinose, and L-rhamnose [10]. One major bioactive compound present in extract solution from different parts of plant was used to propose the mechanism of ZnONPs formation. For the leaves extract, a vitamin (L-ascorbic acid) was chosen, a flavonoid (quercetin) for the flowers, and a fatty acid (oleic acid) for the seeds. The nitrate ion ionizes the bioactive chemicals first via these pathways, and subsequently the resultant cations engage with Zn^2+^ via electrostatic attractions. A follow-up study is required to look at the possibilities of controlling the shape or growth of NPs. [84].

**Fig. 6:**
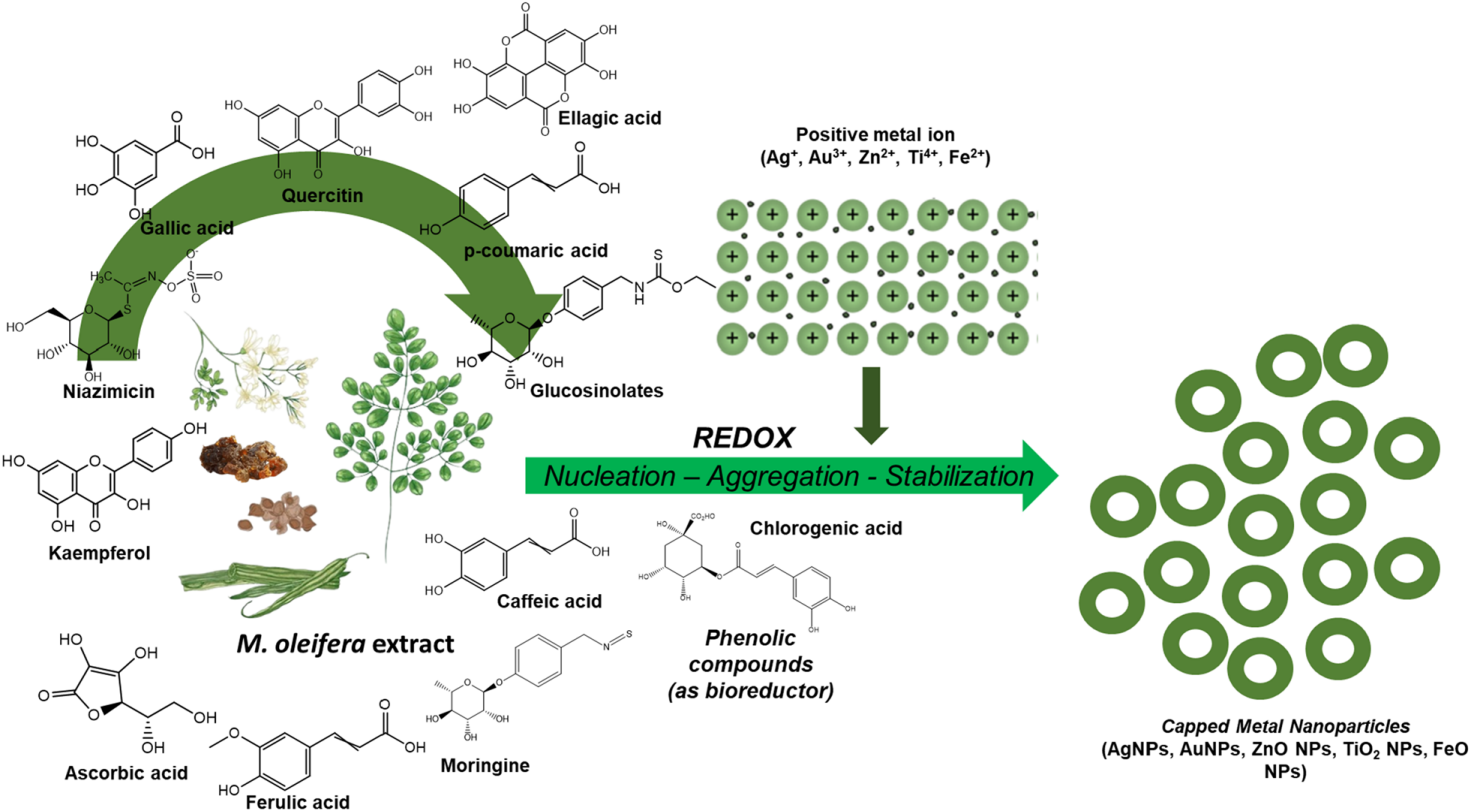
Incorporation of phenolic compounds in the *M. oleifera* NP synthesis

In short, among the major constituents of *M. oleifera* extract, the participation of polyphenols from *M. oleifera* extract in the synthesis of NPs was proved (Fig. 6).

## Applications of *M. oleifera* NPs

**Fig. 7:**
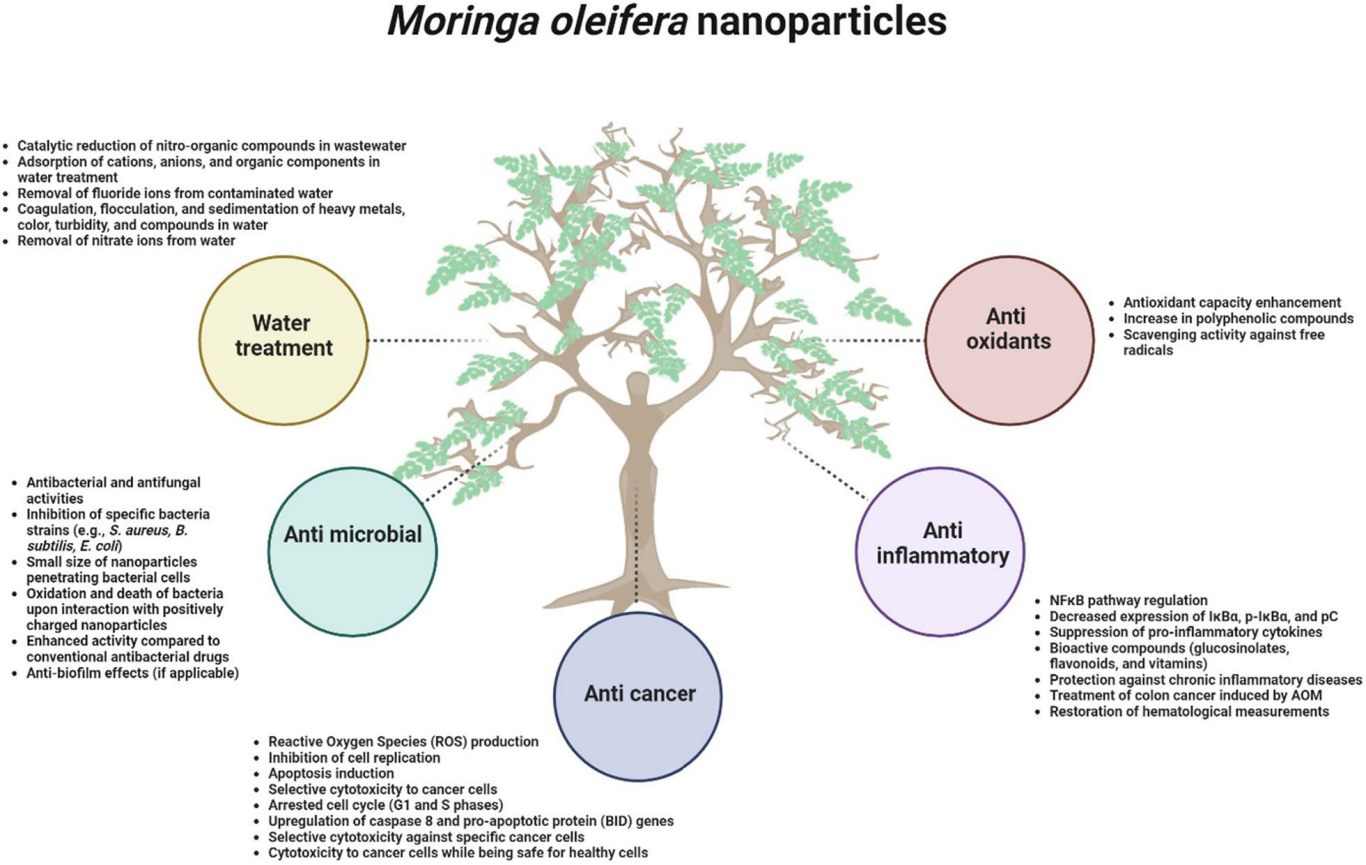
Several mechanisms of function from green synthesized metal NPs utilizing *M. oleifera* extract

Several applications of *M. oleifera* NPs have been extensively studied (Fig. 7). In this section, we will discuss each application in detail.

### Anticancer

A successful synthesis of AgNPs using *M. oleifera* stem bark extract showed excellent anticancer activity against HeLa cells. The anticancer activity of AgNPs is through producing high amount of reactive oxygen species (ROS), inhibition of cell replication and apoptosis induction. Thus, these AgNPs can be suggested as a potential agent for cancer therapy [90]. The leaves mediated AgNPs enhanced antioxidant capacity, concentration of polyphenolic compounds, reducing power and scavenging activity against free radicals, and increased cytotoxicity to cancer cells due to presence of phenolic compounds [33]. One pot synthesis of AgNPs using ethanolic extract of *M. oleifera* produced AgNPs with selective cytotoxicity to leukemia cells (CD3 +) cells but non-toxicity to normal myeloid cells. The mechanism of function was through arrested cell cycle at G1 and S phases, and induced cell apoptosis by upregulation of *caspase 8* and pro-apoptotic protein, *BID*, genes [13]. *M. oleifera* leaf extract-directed synthesis of AgNPs showed selective cytotoxicity against acute myeloid leukemic cells and did not significantly affect the viability of healthy CD34 + cells. The AuNPs, synthesized using the aqueous flower extract of *M. oleifera,* were cytotoxic to the lung cancer cells whereas non-toxic to normal healthy cells. AgNPs synthesized using *M. oleifera* extract were both cytotoxic and genotoxic to breast cancer cells while being safe for healthy human endothelial cells [33]. Similarly, 25 mm sized, spherical shaped, and green-synthesized NPs from *M. oleifera* leaf aqueous extract was investigated for their anti-cancer properties in human colon cancer cell lines such as SW480 and HTC116. As a result, there was a decreased expression of *CTNNB1* and *LRP6*, and increased *LRP5* transcript [91]. A comparative study was conducted on one-step synthesis of silver (Ag), gold (Au) and Ag/Au bimetallic nanoparticles using aqueous leaf extract of *M. oleifera* and their cytotoxic effect against hepatocellular carcinoma (HepG2) and breast cancer (MDA-MD-231 and MCF-7). Compared to all the metal nanoparticles tested, AuNPs have shown to possess higher cytotoxicity with IC_50_ value of 37.22–49.94 µg/ml compared to Ag/Au bimetallic nanoparticles. However, AgNPs did not show cytotoxicity up to 60 µg/ml indicating that no synergistic effect was found when using bimetallic nanoparticles [92]. In summary, using right combination of nanoparticles mixture is crucial to exhibit anti-cancer properties.

### Anti-inflammatory

Previous review described that the green synthesized NPs from *M. oleifera* have also been shown to have anti-inflammatory properties. The NPs regulate the NFκB pathway by decreasing the expression of IκBα, p-IκBα, and pC. The reduction of these proteins may lead to the suppression of pro-inflammatory cytokines. Additionally*, M. oleifera* contains bioactive compounds such as glucosinolates, flavonoids, and vitamins, which have potent antioxidant and anti-inflammatory activities, offering protection against chronic inflammatory diseases [65]. Another research article investigated the potential of *M. oleifera* leaves extract incorporated with Ag-NPs in treating colon cancer induced by AOM (azoxymethane), which is a carcinogenic compound that is commonly used to induce colon cancer in animal models for research purposes. The extract was able to restore hematological measurements to normal levels in both simultaneous and post-treated groups, suggesting its potential anti-cancer properties. However, the specific mechanism behind these properties was not discussed in detail [93]. A recent study compared green silver oxide nanoparticles (AgONPs) synthesized using *M. oleifera* stem extract with those of standard pharmacological drug, diclofenac sodium. At a concentration of 80 µg mL^-1^, synthesized NPs showed significant anti-inflammatory effects compared to that of standard drug suggesting the use of green methods for the treatment of inflammation related disorders [94]. Further studies need to be conducted to reveal the anti-inflammatory effects of metal nanoparticles synthesized using *M. oleifera* extracts. This will help medical professionals to wisely choose drugs for treating inflammation without major side effects.

### Antimicrobial (Antibacterial, antifungal, and antiviral)

Several reports showed potential use of NPs from Moringa extract as the antimicrobial substance (Fig. 10). The production of AgNPs utilized *M. oleifera* flower extract contains natural antioxidants capable of producing antibacterial compounds against *K. pneumonia* and *S. aureus* [95]. Antibacterial activity of *M. oleifera* flower extract AgNPs was shown against various pathogens namely *S. aureus*, *B. subtilis*, *M. luteus*, *S. paratyphi*, *P. aeruginosa*, and *K. pneumoniae* [96].

The antibacterial activity of *M. oleifera* leaves AgNPs with zone of inhibition (ZOI) in the range of 6–15 nm was shown for different kinds of bacteria namely *S. aureus*, *C. tropicalis*, *C. krusei*, and *K. pneumonia* [97]. The antimicrobial activity is due to the action of *M. oleifera* extracts as the antimicrobial agent that reduces the metabolic activity of different pathogens [96]. The AgNPs derived from the leaf extract of *M. oleifera* showed strong inhibition of both gram-negative and gram-positive bacteria as well as various fungal species irrespective of fresh and freeze-dried leaf sample used. In fact, the variation in membrane stability of the bacteria has no effect on the mode of action of these AgNPs, because gram-positive bacteria have a thick peptidoglycan layer, whereas gram-negative bacteria have a hard outer membrane structure made of lipids and lipoproteins [15]. The biosynthesis of colloidal nanosilver using *M. oleifera* leaf extract as a reducing agent was found to have strong inhibitory action against several types of bacteria, including *E. coli*, *S. aureus*, *S. typhi*, and *B. subtilis*. This inhibitory action was attributed to the smaller size NPs that were synthesized, which were found to change the local electronic structure on their surface. This change in electronic structure enhanced their chemical reactivity and bactericidal effect, allowing them to interact with bacteria at a molecular level and disrupt their cellular processes, ultimately leading to their inhibition or death. These findings suggest that biosynthesized AgNPs could be used as an effective antimicrobial agent in various applications, including packaging materials [98].

The biosynthesized TiO_2_ NPs also showed the ability to improve resistance in the wheat plants against *Bipolaris sorokiniana*, which is responsible for spot blotch disease [72]. 40 mg/L foliar application of biogenic TiO_2_ NPs from Moringa extract induced disease tolerance in wheat plants [72]. The potential use of formulated FeONPs from Moringa extract was shown as an antimicrobial substance. Jegadeesan et al. reported that the biogenic Fe NPs made from Moringa leaves extract possessed antimicrobial activity against *S. aureus* and *B. subtilis* [78]. Other study reported that the antibacterial effect of FeONPs from Moringa seeds and leaves extract was superior on gram negative strain compared to different antibiotics (Ampicillin, Gentamycin, Erytomycin and Vancomycin), at 20 μg/mL of concentration [43]. Moreover, combination with other plant extract in addition to Moringa showed superior antibacterial activity against pathogens such as *S. aureus, E. coli, Shigella,* and *S. typhi*, at 10 μg/mL of concentration [75]. In concurrence with that, FeO-NRs also inhibited the growth of *S. aureus*, *P. aeruginosa*, *E. coli*, *Shigella*, *S. typhi*, and *P. multocida* with a higher activity at low concentration [76]. NPs activity against microorganisms occurs due to their small size which can penetrate the bacterial cell [78]. Mirza et al. [99] reported that when the negatively charged microorganism electrostatically interacts with positively charged FeONPs, they oxidize and die. It is worth noting that, when compared to traditional antibacterial medications, the bacterial strains exhibit robust and effective susceptibility to the manufactured Fe or FeONPs at lower concentrations [43, 75, 76, 78].

The synthesized ZnONPs from Moringa leaves extract were studied for antibacterial activities against bacteria like *Bacillus subtilis* and *E. coli* at a very low concentration [83]. Similar results also reported from Moringa roots extract based ZnONPs that exhibited anti-bacterial activities against Gram-positive and Gram-negative bacteria, *B. subtilis and E. coli* [38]. Moreover, ZnONPs made from Moringa crude gum exhibited excellent antibacterial activity against *E. coli* and *S. aureus*, while good activity was observed against “super bug” methicillin-resistant *S. aureus* (MRSA) [10]. Studies indicate that silver (Ag) and ZnONPs prepared by biosynthetic techniques utilizing Moringa leaf extract have enhanced antibacterial properties compared to the same NPs prepared using other agents [100].

The hydroalcoholic extract of *M. oleifera* leaves was successfully used for the production of Cu NPs of < 50 nm exhibiting antioxidant, anti-bacterial, and anti-fungal activities. Bismuth nanoparticles produced from the hydroalcoholic extract of *M. oleifera* leaves showed antioxidant, antifungal, and antibacterial activities [41]. AuNPs derived from *M. oleifera* extract were less cytotoxic and aided in the regeneration of neural cells in vivo [101]. Similarly, ZnO NPs synthesized using *M. oleifera* leaves prevented neuroendocrine toxicity from rotenone by regulating acetylcholinesterase activity and oxidative stress [102].

### Antioxidants

Phenolic compounds such as flavones, flavonols, phenolics, phenolic acids, tannins, and proanthocyanidins derived from the shikimate, pentose phosphate, and phenylpropanoid pathways are the major secondary metabolites which confer antioxidant characteristics of moringa. The use of *M. oleifera* leaf extract in the synthesis of AgNPs is rapid, eco-friendly, non-toxic, economical, and single step procedure and highly efficient than standard pentavalent antimonial treatment against *Leishmania major* infection probably by boosting antioxidant activity [34]. Better radical scavenging activity against 2,2-diphenl-1-picrylhydrazyl (DPPH) shown by rGO-TiO2 (phytoreduced graphene oxide-titanium dioxide) compared to GO, rGO and TiO_2_ separately. In this research article, *M. oleifera* stick extract is used as a greener reducing agent in the synthesis of rGO-TiO2 nanocomposites. The neutralization reaction of DPPH's free radical nature causes antioxidant performance, with electron transit between reactants showing rGO-TiO_2_ as an efficient antioxidant [73]. The antioxidant properties of Moringa were also greatly influenced by environmental parameters such as annual precipitation, minimum temperature, and maximum temperature on soil. Therefore, a recent study was conducted to determine the antioxidant properties of phenolic content in NP *M. oleifera* [103]. Therefore, studying the antioxidant capabilities of *M. oleifera* NPs produced using distinct extracts derived from different places is vital.

### Water treatment

**Fig. 8:**
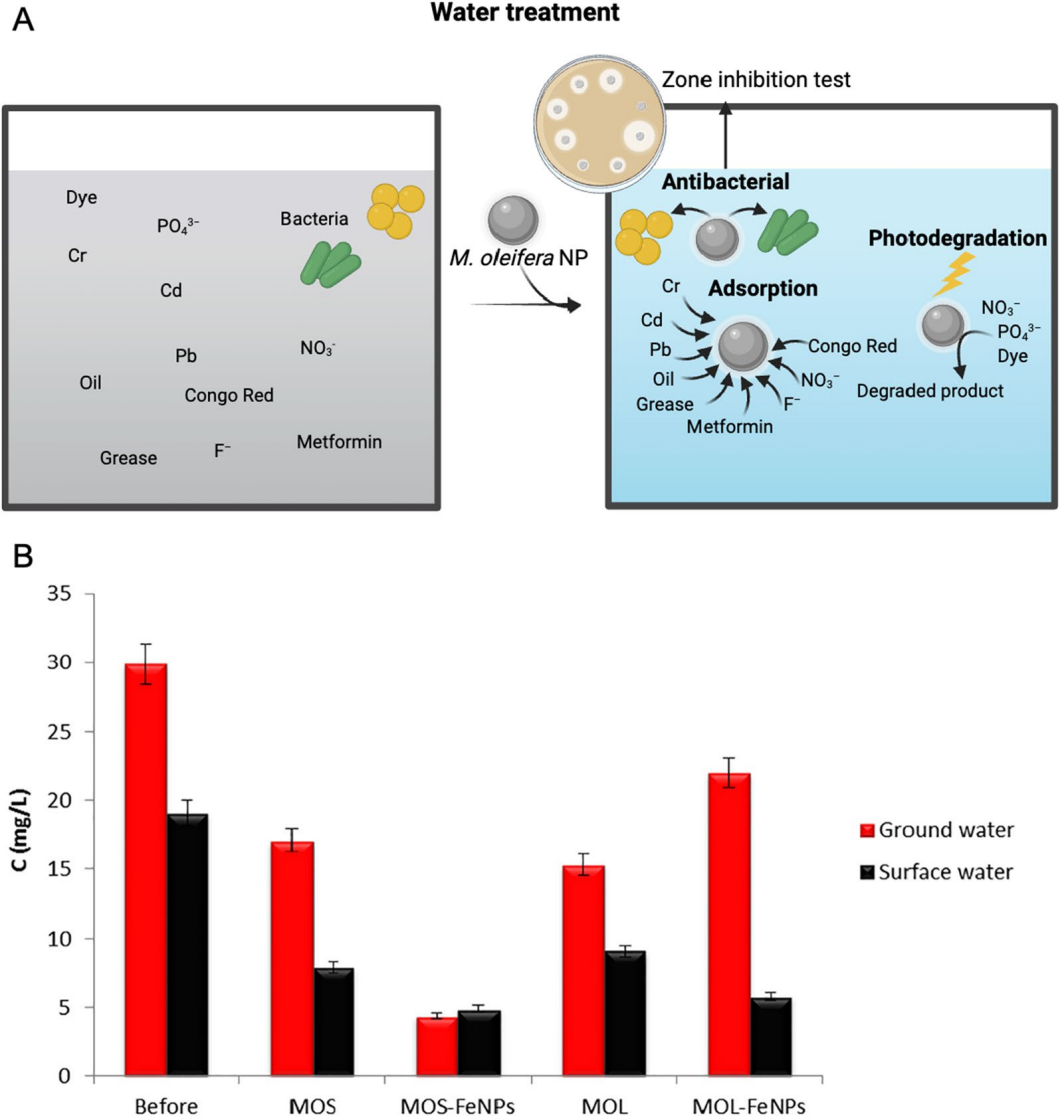
The application of *M. oleifera* derived NPs in water treatment. **A** *M. oleifera* derived NPs could remove the heavy metals, ions, oils, greases, pharmaceuticals using the adsorption capacity. Photodegradation activity of *M. oleifera* derived NPs towards dyes, nitrate, and phosphate was shown. **B** NO_3_^−^ removal from surface and ground water using plant extracts and synthesized nanoparticles. *MOL M. oleifera* leaf, *MOS M. oleifera* seed. The original image was adapted with permission from Ref. [43]. 2023, Shadi Rahimi

Moringa extract has been successfully used for the synthesis of various NPs for theragnostic applications (Fig. 8). Anand et al. used petals of *M. oleifera* for synthesizing stable AuNPs using a green chemistry approach. These NPs could catalytically reduce nitroaniline and nitrophenol in the presence of UV light within 3 min which indicates their possible applications in removing nitro-organic compounds from the wastewater [68].

A facile NPs of FeO used in the preparation of different adsorbents for the adsorption of cations, anions, organic components, among other substances present in contaminated water [104]. When tested on fluoride ion removal, ferric oxide NPs synthesized from Moringa extract show spontaneous reaction at 30 °C. Moreover, even though the adsorption capacity of NPs is reduced after first washing, the reuse of the same NPs is likely, which can lead to a cost reduction. The NPs produced in this study are very promising materials for fluoride ion removal due to their adsorption capacity and minimum contact time to reach equilibrium [77].

The functionalized NPs were used in the coagulation/ flocculation and sedimentation (CFS) of surface raw water under the influence of an external magnetic field. A straightforward approach for the manufacture of magnetite (Fe_3_O_4_) and subsequent functionalization by Moringa extract (MO-Fe_3_O_4_) demonstrated substantial removal efficiencies of the assessed parameters, including the removal of heavy metals such as lead (Pb), cadmium (Cd), and chromium (Cr) from water [79] or the removal of apparent color, turbidity, and compounds with UV 254 nm absorption from surface waters. Within 30 min, a magnetically activated *M. oleifera* coagulant can successfully remove 85% of apparent color, 90% of turbidity and 50% of compounds with UV 254 nm absorption in surface waters under the influence of an external magnetic field [27]. Moreover, other report showed removal efficiency of 97.1% for the apparent color, 96.8% for turbidity, and 58.3% for the compounds with absorption at UV 254 nm after 10 min of magnetic sedimentation, proving that the CFS process can be optimized to reduce the sedimentation time from 30 to 10 min while still achieving high removal efficiencies for these contaminants. The reuse tests revealed that the functionalized magnetic NPs may be reused up to two times in a row with no noticeable reduction in efficiency in eliminating the examined parameters. Furthermore, the residual values obtained for the turbidity and apparent color parameters following the method were adequate without the requirement for a subsequent treatment operation, such as filtering [79]. In conclusion, coagulants based on natural ingredients, associated with FeONPs, provide a good alternative to the use of synthetic compounds in water treatment processes [27].

It was also studied whether green synthesized FeONPs from Moringa extract could be used to remove nitrate ion (NO_3_^−^) from surface and ground water [43] (Fig. 8B). NO_3_^−^ removal by MOS-FeNPs was 85% which was higher than that of *M. oleifera* extract. Lower pH of aqueous solution must be maintained for higher percentage of NO_3_^−^ removal, regardless the type of FeNPs used [43]. It has also been reported that zinc nitrate hexahydrate can be effectively chelated, oxidized, or reduced through the biosynthesis of ZnONPs using a natural extract from *M. oleifera* leaves [85]. Application of various types of moringa NPs in water treatment were summarized (Table 2).

**Table 2:** The application of NPs made from *M. oleifera* in water treatment

Particle	Activity	Size (nm)	References
AgNPs from *M. oleifera* seed	Photocatalytic activity toward organic dyes (methylene blue (> 81%), 4-nitrophenol (> 75%), orange red (> 82%) and > 80% of the Pb removal, antimicrobial activity against Gram positive (*Staphylococcus aureus*) and Gram negative (*Escherichia coli*, *Pseudomonas aeruginosa*, and *Salmonella* ent*e*rica *typhimurium*)	4	[14]
AgNPs from *M. oleifera* flower	Antibacterial (*Klebsiella pneumoniae*, *S. aureus*), sensing copper ions from 1 to 12 mM concentrations	22	[4]
FeO-NPs from a mixture of *M. oleifera* leaf extract and *Psidium guajava* leaf extract	Photocatalytic activity toward methylene blue	82	[163]
*M. oleifera* activated carbon modified with chitosan and Fe_3_O_4_ NPs	Cr adsorption capacity of 130.80 mg/g	NA	[164]
Cellulose nanofibers from *M. oleifera*	Cd (> 12 mg/g) and Pb (> 80 mg/g) adsorption	80–160	[165]
Activated carbon from *M. oleifera* seed modified with FeONPs	94.2% oils and greases adsorption	Average pore size 3.1	[166]
Aluminium oxide NPs *M. oleifera* gum activated carbon	Photocatalytic 94% nitrate and 95% phosphate removal	16.12	[167]
*M. oleifera* with Fe_3_O_4_ NPs	Elimination of *S. aureus*	NA	[168]
Ag and CuONPs from *M. oleifera* leaf and stem	Antimicrobial activity against *E. coli, S. typhi, P. aeruginosa*, *Klebsiella variicola*	NA	[169]
Activated carbon from *M. oleifera* seed modified with AgNPs	Methyl blue and methyl orange 90% and 77% photodegradation	200 to 305	[170]
FeONPs from *M. oleifera* leaf and stem	65.01 mg g^−1^ metformin adsorption capacity	NA	[171]
ZnONPs from *M. oleifera* leaves extract	Adsorption of congo red dye reached equilibrium in 120 min	10	[86]

## Factors affecting the NP efficacy using *M. oleifera* extract

### Size

Size of the NPs is the foremost features decide the selection of nanocarriers to be incorporated [105]. NP size affects cellular absorption by influencing the enthalpic and entropic characteristics that determine the adhesiveness of NP on the cell surface [106]. Smaller NPs possess a high likelihood to penetrate the cell membrane in comparison to huge ones. It is also perceived that the NPs with the size of > 50 nm cohered to the outer membrane caused a slight intervention. NPs with the size of 25–35 nm that cohered to the surface gave minimal deformity. Smaller NPs < 10 nm caused distortion by bending the membrane inwards stacked with several NPs [107]. Salomoni et al. (2017) evaluated the antibacterial activity of AgNPs against *P. aeruginosa* and *E. coli*. They stated that, NPs with the size < 100 nm showed better inhibitory action than that of larger NPs, > 1000 nm [108]. CuONPs synthesized from *M. olifera* which had size 12–18 nm showed great antimicrobial activity [109]. AgNPs synthesized from *M. olifera* leaves of size 5–22 nm showed great antibacterial activity against *S. aureus* than *K. pneumoniae* [4]. The AgNPs synthesized from *M. oleifera* leaves which had size of 57 nm showed a great inhibition zone against *S. aureus, C. tropicalis,* and *K. pneumoniae* [97]. The secondary metabolite concentration in the Moringa extract influences the reduction/oxidation and stabilization of NPs, which ultimately determines the size of the NPs.

In addition, the removal and cellular uptake pathway in vivo can also be affected by the NPs size [110]. A study reported that the smaller the NPs, the higher the renal clearance. NPs of size less than 10 nm can effortlessly pass through the blood vessels and get eliminated by kidneys, whereas the larger NPs get arrested by the MPS cells [107]. Liu et al. [111], led an experiment with AuNPs of different sizes. In which the non- phagocytic cells had higher cellular uptake with decreased NPs size (< 10 nm), while the macrophage cells had higher cellular intake with increased NPs size (10 nm).

The NPs size also performs an important role in their extended circulation, bio-distribution, and elimination and permeability due to nanoscale structure [112]. In particular NPs properties, which include thermal, luminescence, electrical, magnetic, permeability, are response to the size of the particles. Altering the pH levels of the synthesis medium can influence the size of NPs since large NPs are synthesized only when pH is acidic [113]**.**

**Fig. 9:**
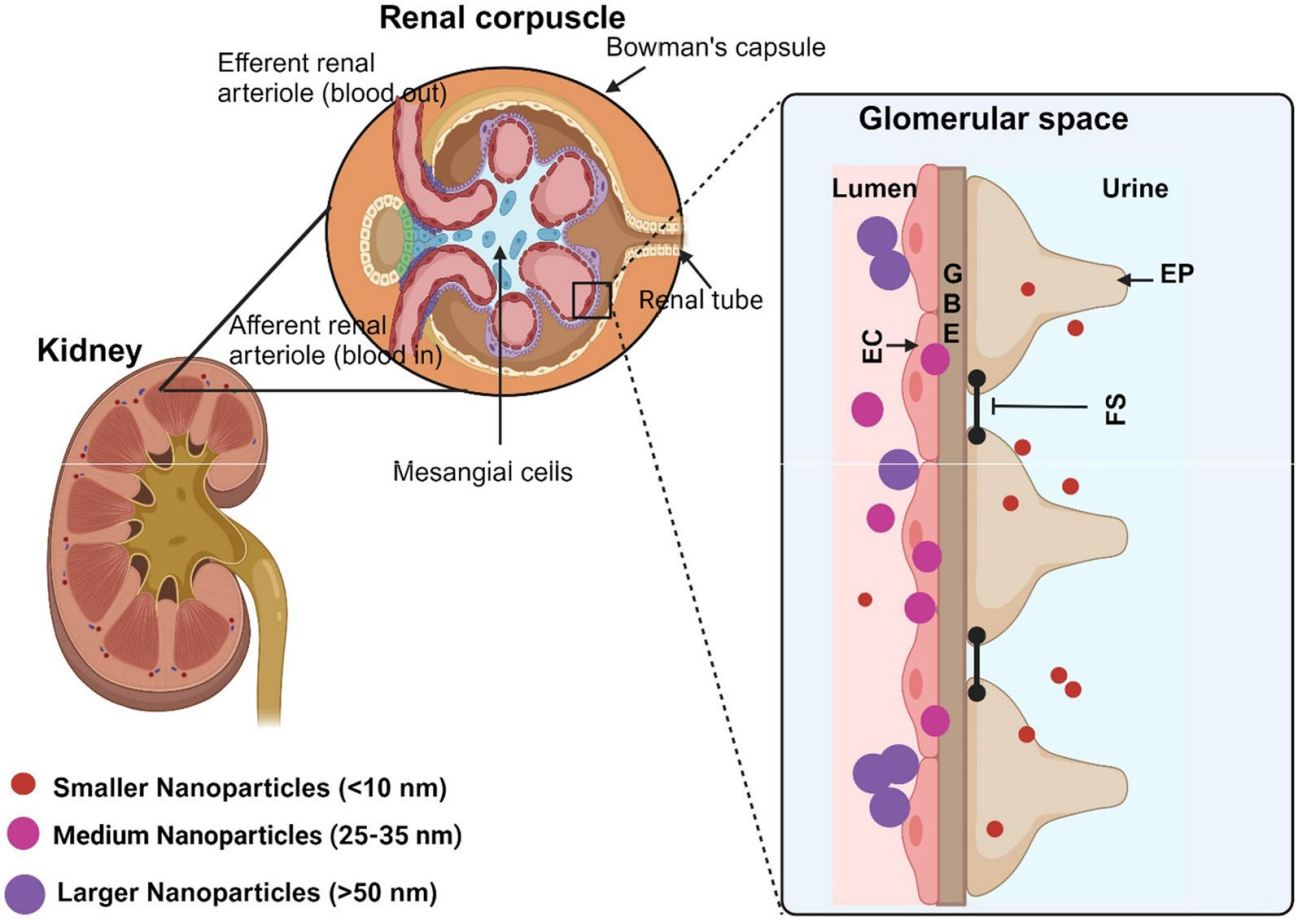
Schematic representation of the kidney showing size dependent elimination. Smaller the NP size, larger the elimination through urine. Medium NPs partially enter, and larger size NPs stacked onto the EC membrane. *EC* Endothelial cells, *GBE* Glomerular basement membrane, *EP* Endothelial podocytes, *FS* Filtration silts

In summary, smaller NPs show better antimicrobial activity, while they can be easily cleared from the body through the kidney (Fig. 9), while larger NPs can be taken by MPS cells.

### Shape

Shape can directly stimulate cell absorption. Choosing the ideal shape for the NPs would facilitate favourable interaction with serum proteins [114]. The study on AuNPs of four different shapes: spherical, rod-shaped, hollow particles and silica shell showed different uptake concentrations for different shapes. The uptake of spherical particles by human endothelial cells was the highest and that of hollow particles [115]. The AgNPs synthesized from *M. oleifera* leaf extract, which was spherical in shape expressed remarkable antimicrobial action, preventing the growth of *S. epidermidis, K. cloacae,* and *E. coli* [61]. The rod and cube shaped AgNPs, showed significant difference of growth reduction for the two studied fungi. The rod shape NPs had less fungi growth compared to the cubic shape [116]. Sphere shaped MnO NPs showed more antioxidant activity than NPs in cubic shape [117].

Studies tend to show that NPs shape and size play a prominent part in the pathways by which particles enter cells, cycling time, identifying the target, and capacity to overcome a biological barrier. The antibacterial activity of triangle-shaped AgNPs was lower than that of spherical particles. Furthermore, as compared to the bigger triangular-shaped AgNPs, the smaller spherical AgNPs were more successful in penetrating inside the bacteria [118]. AgNPs with spherical, triangular plate and disc shapes were experimented and result showed that a high concentration of spherical AgNPs in the ratio was resulted in a significant antibacterial activity [119].

These shape and size are likely to affect how cells recognize and react to the NPs during transport in blood, primarily in small vessels and malignant cells vessels [120]. The investigation in the uptake and transport of different shaped (spheres, rods and discs) NPs across intestinal cells clearly illustrated that rods have greater cellular uptake, while the disc shaped NPs showed greater transport among others [121]. Spheres and rods exhibit different binding patterns in a shear force.

Shape is a core factor that determines the nanomaterials (NMs) delivering drugs efficiently and accurately. The onset of adhesion and prolonged interaction post adhesion are affected by the particle shape [122]. The probability of maintaining adherent or being washed away after initial adhesion, internalisation and drug release activities are determined by the shape of the NPs. Several studies stated that the spherical NPs are better option for delivering drug as they can internalize rapidly than rod-shaped NPs [123]. Shape can also affect where NPs accumulate in the body. For example, spherical and cylindrical NPs accumulate in the liver while disc-shaped NPs accumulate in the heart [124].

**Fig. 10:**
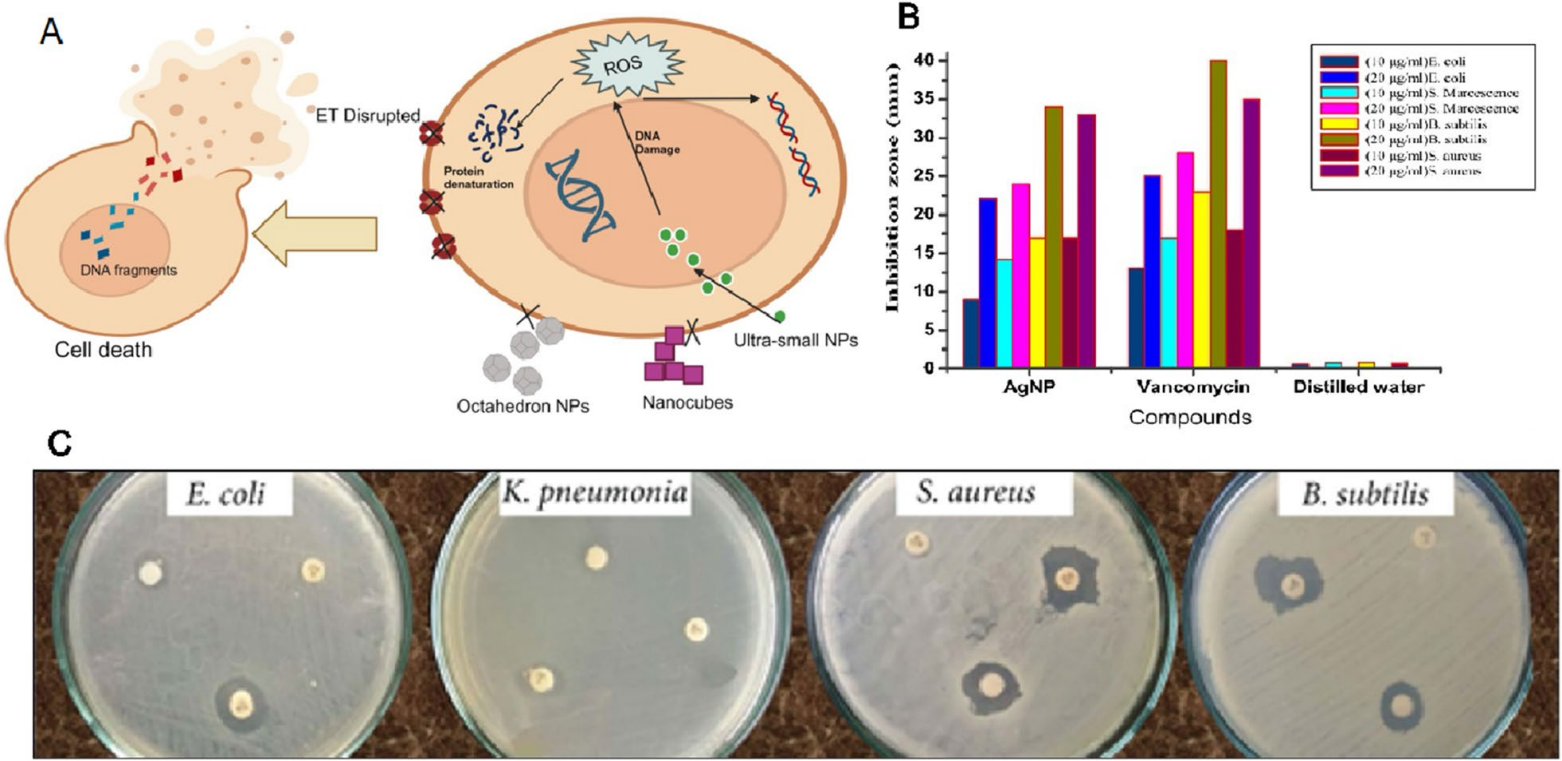
**A** *M. oleifera* NPs with small size and spherical shape penetrate into the bacteria and show strong antibacterial activity. ET, Electron transport; NPs, Nanoparticles; ROS, Reactive oxygen species. **B** Antibacterial activity of spherical *M. oleifera* AgNPs compared to Vancomycin against different strains of bacteria. The original image was adapted with permission from Ref. [159]. 2023, Shadi Rahimi. **C** Antibacterial activity of spherical *M. oleifera* AgNPs. The original image was adapted from Ref. [158]

A concentration dependent increase of FeCl_3_ changed the morphology of the as-prepared moringa seeds samples, and the NMs appears small bulk or sheet shape with smooth surface. On the other hand, a few of folded nanosheets with crumpled surface seen without FeCl_3_ can effectively prevent the nanosheets from stack, improve the open surface area, facilitate the electrolyte ion diffusion in the sheet-to-sheet, and offer more active sites to form electric double layers [125]. In general, the NPs with spherical shape can penetrate the bacteria and showed stronger antibacterial activity compared to NPs with other shapes (Fig. 10).

### Surface chemistry

NPs surface charge has an impact on their solubility, distribution, stability, absorption, and cellular toxicity. According to a series of studies, it is revealed that the positive ions on Ag are critical for their antimicrobial activity. The electrostatic interaction between the negatively charged cell membrane of microorganism and positively charged NPs is most likely what causes this activity [97]. Charge polarity and density both contribute to cytotoxic activity of NP. In nonphagocytic cells, positively charged ZnO, silica, silica-titania hollow and gold NPs are more cytotoxic than negatively charged ones [126]. Modulation of the surface charge on NPs impacts the immune response. Immune cells showed more inflammatory response when exposed to positively charged nanoparticles than when exposed to neutral or negatively charged NPs [127].

Particle surface can have a variety of defects, including atomic vacancies, variations in atomic coordinate, loose bonds, and structural disorder. Such surface imperfections may cause disturbed spins at the surface, which could cause N to become surface magnetised [128].

## Challenges and toxicity profiles associated with bio-synthesized nanoparticles

Nanoparticles synthesized through environmentally friendly methods are acknowledged for their extensive range of biological applications, particularly in serving as an effective mechanism for drug delivery [129, 130]. While nanoparticles derived from *M. oleifera* hold promise for diverse fields such as medicine and agriculture, it is essential to thoroughly assess their biosafety profile and toxicological concerns to ensure their safe application [131]. This recognition stems from their remarkable physicochemical attributes and behavioral properties. Nonetheless, as the utilization of these nanoparticles in biological contexts continues to expand, there exists considerable uncertainty regarding their safety in the human system. Due to their infinitesimal size and distinct characteristics, nanoparticles find frequent application in the realm of nanomedicine and as carriers for pharmaceutical agents. However, potential toxicity towards healthy human cells, tissues, and organs is a significant concern, influenced by factors such as crystallinity, solubility, aggregation, surface characteristics, morphology, surface area, and dosage-dependent attributes [132, 133]. It becomes imperative to investigate the nanoparticles' capacity to elicit an innate immune response. Various nanoparticles, including metal and metal-oxide nanoparticles, have been reported to induce pro-inflammatory effects in both in vitro and in vivo studies [134, 135].

Nanoparticles predominantly enter the human body through the respiratory system, where they often provoke inflammatory responses, primarily driven by redox stress. Adding to the complexity, nanoparticles exhibit a remarkable capability to breach the blood–brain barrier, with the olfactory nerve hypothesized to serve as a conduit for their entry. This raises concerns about the potential impact of nanoparticles on the olfactory mucosa, from where they could traverse to the brain, exerting influence on both its health and functioning [136, 137]. Moreover, oxidative stress emerges as a central and extensively studied consequence of nanoparticle exposure. The heightened production of ROS, surpassing the capacity of antioxidants to neutralize them, establishes an oxidative stress condition [138, 139]. Notably, various by-products of biological reactions, including hydroxyl radical, hydrogen peroxide, peroxynitrite, nitric oxide, and superoxide radical, represent key contributors to ROS generation. The ensuing ROS unleashes havoc on cellular components, causing damage to proteins, lipids, and crucial biomolecules. This cascade of molecular events triggers responses reminiscent of the activation of the nicotinamide adenine dinucleotide phosphate (NADPH) system [140]. Consequently, this disruption cascades through the electron transport chain, induces depolarization of the mitochondrial membrane, and alters the structural integrity of mitochondria, collectively posing significant implications for cellular and, consequently, overall health [133]. For instance, silver nanoparticles (AgNPs) when ingested potentially accumulated in all of the organs tested, and most predominantly distributed in intestine and stomach, skin and exhibits toxicity [141, 142]. In the skin, Ag induces blue-grey discoloration known as argyria. Animal studies indicated that their toxicity depends on dose-dependent exposure, causing death, weight loss, decreased activity, liver enzyme impairment, and immunological consequences [142]. Whereas, in kidney AgNPs resulted in the deposition of Ag in renal glomerular basement membrane [142, 143].

A study conducted by Vijaykumar et al., delineates, for the inaugural instance, the ecologically mindful synthesis of MgO NPs encapsulated within Moringa gum (Mgm-MgO NPs). A comprehensive evaluation was undertaken, encompassing assessments of their antioxidant efficacy, potential for hemolysis, cytotoxicity in human ovarian teratocarcinoma (PA-1) cells, phytotoxicity in mungbean (*Vigna radiata*), antiangiogenic attributes via the chorioallantoic membrane (CAM) chick embryo assay, and in vivo toxicity appraisals employing zebrafish embryos. Remarkably, the Mgm-MgO NPs exhibited notable antioxidant prowess. At a concentration of 500 μg/ml, these nanoparticles induced substantial hemolysis, registering at 72.54%, whereas lower concentrations demonstrated negligible hemolytic effects. The cytotoxic impact on PA-1 cells, evaluated through the MTT assay, revealed a significant reduction in cell viability across a concentration range of 0.1–500 μg/ml of Mgm-MgO NPs. Contrastingly, Mgm-MgO NPs displayed no discernible impact on seed germination but markedly influenced the root and shoot lengths of mungbean. Furthermore, the CAM assay, employed to scrutinize antiangiogenic potential, demonstrated no significant alterations following a 72-h exposure to Mgm-MgO NPs. Concluding the investigative spectrum, the embryotoxicity assay utilizing zebrafish embryos elucidated that Mgm-MgO NPs, within the concentration range of 0.1–500 μg/ml, exhibited no deleterious effects on morphology, mortality, or survival rate [144].

In a study conducted by Hou and his colleagues, the impact of ZnONPs on cellular processes was investigated, revealing disruptions in DNA replication across various phases of the cell cycle pathway, including G_1_, M, and G_2_, alongside the impairment of mini-chromosome maintenance. The cytotoxic effects induced by nanoparticles were found to extend to multiple levels, affecting physicochemical, metabolic, and molecular pathways [145]. The size of NPs emerged as a noteworthy factor, with smaller particles exhibiting larger surface areas, enabling intricate interactions with cellular components such as carbohydrates, fatty acids, proteins, and nucleic acids. This observation suggests that particle size may play a role in influencing the cytotoxic efficacy of NPs. A significant contributor to the observed cytotoxicity was identified as the disturbance of intracellular calcium (Ca^2+^). Despite Ca^2+^ being a pivotal signaling molecule involved in metabolic regulation, its elevated levels were found to induce acute toxicity on cellular mitochondria [146, 147]. This, in turn, triggered apoptosis through mechanisms such as the preferential release of cytochrome c, heightened ROS production, and the opening of the inner mitochondrial pore, ultimately culminating in the demise of the individual cell [148]. Contrastingly by a current investigation conducted by Mahfouz et al., entails the progression of a more reliable methodology for the biofabrication of zinc oxide nanoparticles (ZNPs) utilizing the green synthesis approach, employing *M. oleifera* extract (MO-ZNPs) as a highly efficient chelating agent for acrylamide (AA). The study delves into the impact of AA on glutathione redox dynamics, liver functionality, lipid profile, and zinc residues in Sprague Dawley rats. AA induced an elevation in liver enzymes, hepatosomatic index, and immunohistochemical manifestation of caspase-3 and CYP2E1 expression. Conversely, co-treatment with MO-ZNPs exhibited a stabilizing effect on the gene expression of glutathione-related enzymes, normalization of hepatocellular enzyme levels, and restoration of hepatic tissue microarchitecture. This substantiates the proposition that MO-ZNPs stands as a promising hepatoprotective agent, mitigating AA-induced hepatotoxicity [149].

For instance, recent findings indicate that the cytotoxic effects of nanoparticles extend beyond inducing cell death; they also encompass the suppression of cell growth when cells are arrested in specific cell cycle phases such as G_2_/M, S, or G_0_/G_1_. Cells arrested in the cell cycle either accumulate significant damage, leading to apoptosis, or engage in repair mechanisms. The occurrence of cell cycle arrest is often cell-type specific and dependent on the particular stage of the cell cycle [150]. The primary toxicity of nanoparticles involves their interaction with DNA, while secondary genotoxicity is contributed by the ROS/RNS they produce. In the indirect primary clastogenic pathway, unsaturated aldehydes generated as a consequence of primary lipid oxidation by ROS serve as precursors for the formation of exocyclic DNA adducts. This intricate interplay underscores the multifaceted nature of nanoparticle-induced cytotoxicity, involving both direct interactions with genetic material and the generation of secondary genotoxic effects through oxidative stress pathways [140].

In many cases Moringa NPs have been found to reduce systemic toxicity caused by the effect of other nanoparticles. The primary objective of the current investigation employed by Abu Zeid et al., was twofold: firstly, to employ an environmentally benign approach for synthesizing selenium nanoparticles (SeNPs) utilizing *M. oleifera* leaf extract (MOLE); secondly, to discern and compare the protective attributes of the green-synthesized MOLE-SeNPs conjugate and MOLE ethanolic extract as interventions for mitigating melamine (MEL)-induced nephrotoxicity in male rats. The nephrotoxic impact of MEL was characterized by an array of morphological alterations in the kidneys, coupled with an up-regulated immune-expression of proliferating cell nuclear antigen (PCNA) and proliferation-associated nuclear antigen Ki–67. Administration of either MOLE or MOLE-SeNPs significantly ameliorated MEL-induced renal functional impairments, oxidative stress, histological anomalies, adjustments in the relative mRNA expression of apoptosis-related genes, and the immune-expression of renal PCNA and Ki-67. In conclusion, both the green-synthesized MOLE-SeNPs and MOLE exhibited nephron-protective properties against MEL-induced nephropathy in murine subjects. Remarkably, this study marks the pioneering disclosure of these effects, with a more pronounced impact observed in the MOLE group compared to the green biosynthesized MOLE-SeNPs conjugate group [151].

Numerous studies have extensively explored the integration of nanoparticles (NPs), such as copper oxide, into biomedical platforms [152]. However, there is a potential concern that these nanoparticles may hasten the process of protein oligomerization. A study conducted by Jaragh-Alhadad and Falahati aimed to elucidate the impact of CuONPs on the oligomerization of β1–42 (Aβ1–42) and its associated neurotoxicity. The study uncovered crucial insights into the adverse effects of CuONPs on central nervous system proteins, which promote the formation of cytotoxic oligomers [153].

Understanding the toxicity of nanoparticles is integral to developing improved and more efficient nanomaterials. Consequently, extensive research is underway to enhance our overall understanding of the effects that nanoparticles exert on the environment and public health. This concerted effort not only seeks to mitigate potential risks but also aims to propel the development of safer materials with broader applications.

## Future perspectives and conclusion

In conclusion, the synthesis of NPs using *Moringa oleifera* extract stands as a remarkable testament to the plant's versatility and potential applications in nanotechnology. The diverse secondary metabolites, notably polyphenols, within *M. oleifera* have been identified as pivotal players in the synthesis process, influencing the size, shape, and biological efficacy of the resulting NPs. The distinctive roles played by these plant components, acting as reducing, stabilizing, and capping agents, have been elucidated, revealing the intricate interplay of biological molecules in the nanomaterial synthesis.

Currently, several studies are focusing on the translational clinical research of nanoparticles synthesized using Moringa extract. Some of them have shown promising effects in *vivo* for treating Leishmaniasis and Retinoblastoma [34, 154]. However biosynthetic techniques have limited control over the particle size, and this may be achieved by better control of temperature, and also adjusting the concentration of *Moringa oleifera* leaf extract. Apart from flavonoids and polyphenols, the role of alkaloids which are mainly cyclic nitrogen compounds (> 12000) may also be evaluated thoroughly. Although several in vitro studies show the antimicrobial and antioxidant potential of nanoparticles synthesized using moringa extract, there is limited in vivo data which poses considerable hurdles in the translation of nanoparticles synthesized using Moringa extract. Furthermore, there is a major gap in the pharmacokinetics and underlying mechanisms responsible for cytotoxicity. Additionally, the optimal size and morphology necessary to elicit a favorable therapeutic outcome must be assessed. PEGylation has been successfully utilized to improve the bioavailability of nanoparticles synthesized using moringa extract [155]. However, studies have also shown that phenolic compounds present in the Moringa extract can also serve as excellent capping agents [156] which may enhance the bioavailability and circulation, although it warrants further studies.

In essence, this comprehensive review underscores the multifaceted potential of *M. oleifera-*synthesized NPs in diverse biomedical applications. As we continue to unravel the intricacies of NP synthesis and their interactions with biological systems, the prospect of leveraging *M. oleifera* as a green tool for nanotechnology holds immense promise. Future research endeavors may focus on refining synthesis methods, optimizing NP characteristics, and elucidating specific molecular pathways for enhanced therapeutic outcomes. The convergence of plant-based synthesis and nanotechnology opens a captivating avenue for advancing bio-inspired materials with unprecedented functionalities, propelling us towards a future where *M. oleifera* nanoparticles play a pivotal role in transformative biomedical solutions.

## Data Availability

Data will be made available upon request.

## Abbreviations

MPS: Mono nuclear phagocyte system
PEG: Polyethylene glycol
TTIP: Titanium tetra isopropoxide
HRTEM: High-resolution transmission electron microscopy
FT-IR: Fourier-transform infrared spectroscopy
AOM: Azoxymethane
ZOI: Zone of inhibition
rGO-TiO2: Phytoreduced graphene oxide-titanium dioxide
MRSA: Methicillin-resistant *S. aureus*
rGO-TiO2: Phytoreduced graphene oxide-titanium dioxide
DPPH: 2,2-Diphenl-1-picrylhydrazyl
